# Role of VapBC12 Toxin-Antitoxin Locus in Cholesterol-Induced Mycobacterial Persistence

**DOI:** 10.1128/mSystems.00855-20

**Published:** 2020-12-15

**Authors:** Sakshi Talwar, Manitosh Pandey, Chandresh Sharma, Rintu Kutum, Josephine Lum, Daniel Carbajo, Renu Goel, Michael Poidinger, Debasis Dash, Amit Singhal, Amit Kumar Pandey

**Affiliations:** a Mycobacterial Pathogenesis Laboratory, Translational Health Science and Technology Institute, Faridabad, Haryana, India; b CSIR-Institute of Genomics and Integrative Biology, New Delhi, India; c Singapore Immunology Network, Singapore; d Drug Discovery Research Center, Translational Health Science and Technology Institute, Faridabad, Haryana, India; e Department of Life Science, ITM University, Gwalior, Madhya Pradesh, India; Delta Stewardship Council

**Keywords:** cholesterol, host-pathogen interactions, mycobacteria, toxin-antitoxin

## Abstract

The worldwide increase in the frequency of multidrug-resistant and extensively drug-resistant cases of tuberculosis is mainly due to therapeutic noncompliance associated with a lengthy treatment regimen. Depending on the drug susceptibility profile, the treatment duration can extend from 6 months to 2 years. This protracted regimen is attributed to a supposedly nonreplicating and metabolically inert subset of the Mycobacterium tuberculosis population, called “persisters.” The mechanism underlying stochastic generation and enrichment of persisters is not fully known. We have previously reported that the utilization of host cholesterol is essential for mycobacterial persistence. In this study, we have demonstrated that cholesterol-induced activation of a RNase toxin (VapC12) inhibits translation by targeting proT tRNA in *M. tuberculosis*. This results in cholesterol-specific growth modulation that increases the frequency of generation of the persisters in a heterogeneous *M. tuberculosis* population. Also, a null mutant strain of this toxin (Δ*vapC12*) demonstrated an enhanced growth phenotype in a guinea pig model of *M. tuberculosis* infection, depicting its role in disease persistence. Thus, we have identified a novel strategy through which cholesterol-specific activation of a toxin-antitoxin module in *M. tuberculosis* enhances persister formation during infection. The current findings provide an opportunity to target persisters, a new paradigm facilitating tuberculosis drug development.

**IMPORTANCE** The current TB treatment regimen involves a combination of drugs administered for an extended duration that could last for 6 months to 2 years. This could lead to noncompliance and the emergence of newer drug resistance strains. It is widely perceived that the major culprits are the so-called nonreplicating and metabolically inactive “persister” bacteria. The importance of cholesterol utilization during the persistence stage of *M. tuberculosis* infection and its potential role in the generation of persisters is very intriguing. We explored the mechanism involved in the cholesterol-mediated generation of persisters in mycobacteria. In this study, we have identified a toxin-antitoxin (TA) system essential for the generation of persisters during *M. tuberculosis* infection. This study verified that *M. tuberculosis* strain devoid of the VapBC12 TA system failed to persist and showed a hypervirulent phenotype in a guinea pig infection model. Our studies indicate that the *M. tuberculosis* VapBC12 TA system acts as a molecular switch regulating persister generation during infection. VapBC12 TA system as a drug target offers opportunities to develop shorter and more effective treatment regimens against tuberculosis.

## Abstract (precis)

The current TB treatment regimen involves a combination of drugs administered for an extended duration that could last for 6 months to 2 years. This could lead to noncompliance and the emergence of newer drug resistance strains.

## INTRODUCTION

Globally, a third of the human population is infected with Mycobacterium tuberculosis, the causative agent of tuberculosis. Being an obligate intracellular pathogen, *M. tuberculosis* has coevolved with humans for centuries (1–3). Interestingly, while in most bacterial diseases the standard therapeutic regimen involves a single antibiotic treatment lasting for a few days to weeks, tuberculosis treatment requires administration of multiple drugs for a duration that could extend from 6 months to 2 years depending on the antibiotic susceptibility profile of the infected strain. This phenotype is attributed to a slow-growing metabolically altered subset of the heterogeneous *M. tuberculosis* population called persisters (4, 5). These persisters are refractory to antimycobacterial drugs and can only be targeted using a strict regimen consisting of a combination of drugs for an unusually extended period. As a result, a protracted regimen thus impels noncompliance and results in an increase in the frequency of multidrug-resistant and extensively drug-resistant tuberculosis cases (6–9).

Although several studies have described the stress-induced generation of persisters under *in vitro* growth conditions (10–13), the exact conditions triggering the generation and enrichment of persisters inside the host during a normal course of mycobacterial infection remain unclear. Upon infection, *M. tuberculosis* induces the formation of lipid-rich foamy macrophages. Lysis of these macrophages results in the formation of the caseous core of a typical “tuberculous granuloma,” providing *M. tuberculosis* with a lipid-rich niche. While residing in this nutrient-deprived granuloma, *M. tuberculosis* adapts itself to utilize host-derived lipids, including cholesterol as a favored carbon source (14). Surprisingly, *M. tuberculosis* does not rely on cholesterol as the sole carbon source during infection (15, 16); nonetheless, this cholesterol utilization causes inhibition of growth and activation of pathways, leading to the generation of persisters in the mycobacterial population (14, 17). *M. tuberculosis* facilitates the intracellular accumulation of cholesterol by upregulating cholesterol biosynthesis pathways and helps to convert resident macrophages into foamy macrophages (18). These findings imply that by hijacking host pathways *M. tuberculosis* creates a favorable niche inside the host, facilitating long-term disease persistence (19, 20).

Toxin-antitoxin (TA) proteins play a crucial role in generating persisters in several bacterial species (21–23). These TA systems, known to modulate growth under various growth and stress conditions, are found in a wide range of bacterial and archaeal chromosomes and plasmids (24–26). Research conducted during the past decade has demonstrated that TA loci act as effectors of dormancy and persistence in several bacterial species (21, 22). Each TA locus consists of genes expressing a TA pair. Antitoxin, being more labile, degrades under specific growth and stress conditions, resulting in the activation of the cognate toxin. The activated toxin modulates growth by targeting growth-related genes.

The genome of *M. tuberculosis* constitutes 88 TA systems, whereas the saprophytic soil-dwelling Mycobacterium smegmatis genome has only one TA locus, clearly highlighting the role of TA systems in bacterial adaptation and survival in a very hostile environment inside the host (27). Based on the mechanism of toxin activation, the TA system is classified into seven different types. The VapBC family, the most characterized of all, belongs to the type II group. The toxin from the type II group targets all forms of RNA, including mRNA, rRNA, and tRNA. Although the type II TA system has been shown to regulate growth in several bacterial species, the exact mechanism regulating the generation of persistence is not well defined. It is hypothesized that each TA pair is required for the survival of bacteria under specific growth and stress conditions (28, 29); however, the presence of a very high number of the TA systems in the *M. tuberculosis* genome also increases the chances of redundancy and the possibility of multiple TA systems regulating specific growth conditions.

In the present study, we identified the role of one such *M. tuberculosis* RNase toxin, VapC12, to be critical for the cholesterol-induced generation of antibiotic persistence in mycobacteria. Our data conclusively demonstrate that cholesterol activates the RNase toxin by disrupting its binding to the cognate antitoxin VapB12. We further demonstrated that the proT tRNA of *M. tuberculosis* is a *bona fide* substrate of the VapC12 RNase toxin and that the toxin-mediated modulation of the proT tRNA regulates the generation and enrichment of the cholesterol-induced persisters in mycobacteria. Finally, we also demonstrated that the *vapC12* gene is critical for *M. tuberculosis* to persist in a guinea pig model of tuberculosis infection.

## RESULTS

### The *vapC12* gene is essential for cholesterol-specific growth modulation in *M. tuberculosis*.

**FIG 1:**
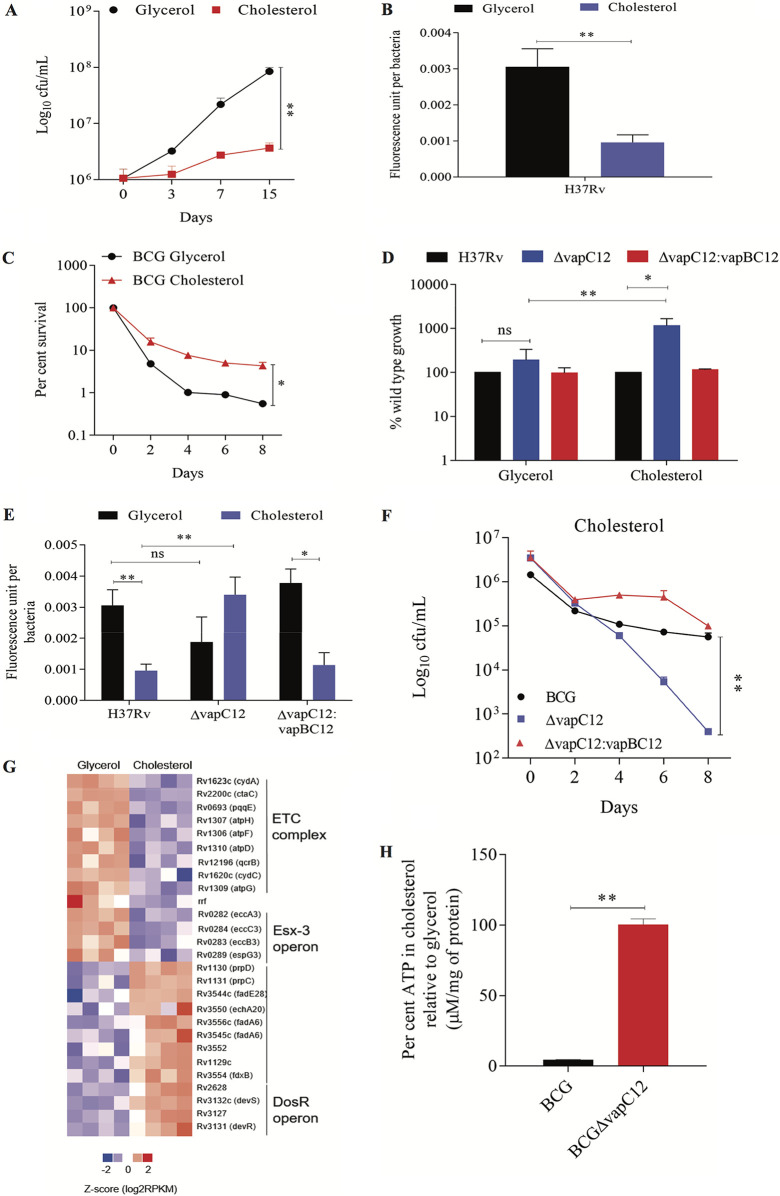
The *vapC12* gene is essential for cholesterol-specific growth modulation in M. tuberculosis. (A) Growth curve of H37Rv in a minimal medium supplemented with 0.1% glycerol and 0.01% cholesterol. Log-phase cultures of H37Rv grown in 7H9 media enriched with OADC were washed with PBS-tyloxapol and resuspended in respective media at an absorbance of 0.005. Growth was estimated by CFU plating on 7H11+OADC plates at different time points postinoculation. Experiments were performed in triplicates, and data represent the means ± the standard errors of the mean (SEM). (B and E) Resazurin-based estimation of the metabolic activity of H37Rv (B) and *ΔvapC12* and *ΔvapC12:vapBC12* (E) strains grown in minimal medium supplemented with glycerol and cholesterol. Strains were serially diluted in a 96-well plate in respective media. The experiment was performed in two independent sets, and the plate was incubated at 37°C for 5 days. One set of the experiment was used for recording fluorescence after adding the PrestoBlue reagent at 570 or 585 nm, whereas the other set was used for the enumeration of bacteria present in each well. The metabolic activity calculated for each well is representative of the mean fluorescence readout per bacteria from three independent experiments. Data were analyzed using unpaired Student *t* test. *, *P* < 0.05; **, *P* < 0.01. (C and F) Kill curve of M. bovis BCG (C) and BCGΔ*vapC12* and BCG Δ*vapC12*:*vapBC12* (F) strains grown in glycerol- and cholesterol-rich media. Log-phase cultures of strains were washed with PBS-tyloxapol and inoculated at an absorbance of 0.05. The cultures were allowed to grow for 4 days before being treated with 5× MIC of rifamycin. Bacterial enumeration was performed through CFU plating of cultures on 7H11+OADC plates at various time points. The kill curve was plotted by calculating the percent survival. The experiment was repeated three times, and data represented as means ± the SEM. Data were analyzed using unpaired Student *t* test. *, *P* < 0.05; **, *P* < 0.01. (D) Percent wild-type growth of the *vapC12* mutant and *ΔvapC12:vapBC12* strains in a minimal media containing 0.1% glycerol and 0.01% cholesterol. Growth was estimated by CFU plating of cultures on 7H11+OADC plates at 8 days postinoculation. The experiment was repeated three times, and the data represent the means ± the SEM. The data were analyzed using unpaired Student *t* test. *, *P* < 0.05; **, *P* < 0.01. (G) Heatmap visualization of differentially expressed transcripts in wild-type H37Rv grown in glycerol and cholesterol media, analyzed by RNA-seq. Expression data of the respective genes based on the adjusted FDR are depicted in the heatmap. The RNA for sequencing was isolated from four different sets of cultures grown in the respective media. (H) Percent estimation of ATP in wild-type BCG and *ΔBCGvapC12* strains grown in a cholesterol-rich media relative to glycerol media. ATP estimated in micromolar concentrations was normalized with per milligram of protein in each sample. The experiment was repeated three times, and the data plotted represent means ± the SEM. The data were analyzed using an unpaired Student *t* test. *, *P* < 0.05; **, *P* < 0.01.

We have previously demonstrated that the utilization of host cholesterol as a carbon source is essential for maintaining persistence during M. tuberculosis infection (14). In this study, we used *M. tuberculosis* grown in minimal medium with cholesterol as the only carbon source (this will be referred to as cholesterol medium henceforth) as an *in vitro* model to examine the role of cholesterol in the formation, maintenance, and enrichment of persisters during *M. tuberculosis* infection. We have initially analyzed the metabolic and replication rates of *M. tuberculosis* grown in the cholesterol media. In comparison to glycerol, we observed a 10-fold decrease in the growth and a 3-fold decrease in the metabolic rates of wild-type (WT) *M. tuberculosis* (H37Rv) grown in the cholesterol media (Fig. 1A and B; see also Fig. S1A in the supplemental material). Surprisingly, the presence of cholesterol was found to have an inhibitory effect on the growth of both M. tuberculosis and M. bovis BCG (BCG) strains (see Fig. S1A to C). An *in vitro* time-kill curve assay revealed a cholesterol-specific increase in the frequency of the generation of a rifamycin-tolerant subpopulation in WT H37Rv (Fig. 1C). Since the TA loci across bacterial species are known to modulate growth (22, 30–32), we speculated the aforementioned phenotype to be regulated by one of the *M. tuberculosis* TA loci. For that, we analyzed the data of a transposon mutagenesis screening performed in *M. tuberculosis* H37Rv to identify genes essential for growth under the condition where cholesterol is provided as the sole carbon source (33). Through manual curation of the data, we identified transposon insertions in six VapC toxins that imparted the insertion mutant strains a significant growth advantage in cholesterol relative to glycerol media. This finding suggests the role of one or all of these toxins in the cholesterol-mediated growth modulation of *M. tuberculosis* (see Fig. S2A). Of these six *vapC* genes, we generated clean deletion mutants for the top two toxins, VapC8 (Rv0665) and VapC12 (Rv1720c), which demonstrated the highest increase in the growth rate. We found that in comparison to the WT strain, the *vapC8*-null strain showed no significant growth difference in the cholesterol media (see Fig. S2B), whereas the mutant strain lacking the *vapC12* gene failed to slow down its growth and was found to be metabolically more active in the cholesterol media relative to the glycerol, suggesting essentiality of this gene in cholesterol-mediated growth retardation. The mutant phenotype was found to be gene-specific because adding back the *vapBC12* locus in the mutant strain restored the WT phenotype (Fig. 1D and E). As expected, the cholesterol-mediated increase in the frequency of the generation of the rifamycin-tolerant population, observed in the WT strain, was reversed in the *vapC12*-null strain, underscoring the role of this putative toxin in the generation of cholesterol-induced antibiotic persistence in mycobacteria (Fig. 1F; see Fig. S3A in the supplemental material). To gain additional insights, we performed transcriptional profiling of these strains in media containing glycerol and cholesterol as a sole carbon source through transcriptome sequencing (RNA-seq) analysis. As expected, relative to glycerol, both WT and *vapC12* mutant strains grown in cholesterol had higher levels of expression of genes involved in cholesterol metabolism, the methyl-citrate cycle, and glyoxylate pathways (34, 35). The slowing down of WT *M. tuberculosis* in the cholesterol media can be attributed to lower expression in the transcript levels of genes involved in respiration (e.g., cytochrome and the ATP synthesis pathway genes). Intriguingly, relative to glycerol, we observed significantly lower expression of genes belonging to the *esx3* locus in *M. tuberculosis* grown in cholesterol media, suggesting the possibility of iron-mediated growth modulation in the cholesterol-rich environment (36, 37). In addition, in comparison to the glycerol, we observed an increase in the transcript levels of DosR regulon genes in wild-type *M. tuberculosis* grown in the cholesterol media (Fig. 1G and Table 1; see also Fig. S3B in the supplemental material). Despite the cholesterol-specific differences observed in the growth phenotype between H37Rv and *vapC12* mutants, expression profiling data showed few differentially expressed genes between the samples, suggesting that a posttranscriptional regulation mechanism plays a decisive role in inducing cholesterol-mediated growth regulation in *M. tuberculosis* (Table 2). Since ATP depletion is one of the mechanisms through which bacteria increase their tolerance to antibiotics leading to persistence (21, 38, 39) and our RNA sequencing data also revealed differential expression of ATP synthesis pathway genes, we quantified intracellular ATP levels in glycerol- and cholesterol-grown BCG cultures. Compared to the glycerol, the cholesterol-grown BCG culture demonstrated a 25-fold decrease in intracellular ATP levels. This cholesterol-specific depletion of intracellular ATP depends on the presence of the *vapC12* gene (Fig. 1H).

**TABLE 1:** Differentially expressed genes of H37Rv cholesterol versus glycerol

Gene ID	Up or down	Log_2_-fold change	Functional category
Rv1623c (*cydA*)	Down	−4.173367035	Intermediary metabolism and respiration
Rv1130 (*prpD*)	Up	4.498655002	Intermediary metabolism and respiration
Rv2990c	Down	−2.46505342	Hypothetical protein
Rv1621c (*cydD*)	Down	−6.129680086	Intermediary metabolism and respiration
Rv0280 (PPE3)	Down	−2.682612285	Pe/ppe
Rv0288 (*esxH*)	Down	−2.201997608	Cell wall and cell processes
Rv2200c (*ctaC*)	Down	−1.889979288	Intermediary metabolism and respiration
Rv2628	Up	2.021486436	Conserved hypotheticals
Rv0282 (*eccA3*)	Down	−1.688985396	Cell wall and cell processes
Rv1813c	Up	2.282592255	Conserved hypotheticals
Rv0284 (*eccC3*)	Down	−1.383205517	Cell wall and cell processes
Rv0106	Down	−3.877437264	Conserved hypotheticals
Rv0693 (*pqqE*)	Down	−1.319452755	Intermediary metabolism and respiration
Rv3132c (*devS*)	Up	1.483961765	Regulatory proteins
Rv2590 (*fadD9*)	Up	1.288687433	Lipid metabolism
Rv1997 (*ctpF*)	Up	1.802391097	Cell wall and cell processes
Rv1307 (*atpH*)	Down	−1.389318935	Intermediary metabolism and respiration
Rv1306 (*atpF*)	Down	−2.691093389	Intermediary metabolism and respiration
Rv3127	Up	1.039567778	Conserved hypotheticals
Rrf	Down	−1.325508211	Stable RNAs
Rv1310 (*atpD*)	Down	−1.397824532	Intermediary metabolism and respiration
Rv1548c (PPE21)	Up	2.939297215	PE/PPE
Rv1394c (*cyp132*)	Up	1.930458768	Intermediary metabolism and respiration
Rv1739c	Up	0.903915373	Cell wall and cell processes
Rv2196 (*qcrB*)	Down	−1.390614561	Intermediary metabolism and respiration
Rv1131 (*prpC*)	Up	3.678185003	Intermediary metabolism and respiration
Rv3544c (*fadE28*)	Up	4.273345021	Lipid metabolism
Rv3550 (*echA20*)	Up	6.005298267	Lipid metabolism
Rv2059	Down	−1.978695163	Conserved hypotheticals
Rv1622c (*cydB*)	Down	−3.539384506	Intermediary metabolism and respiration
Rv1303	Down	−2.450539716	Cell wall and cell processes
Rv3131	Up	0.863421873	Conserved hypotheticals
Rv3556c (*fadA6*)	Up	1.938757989	Lipid metabolism
Rv0287 (*esxG*)	Down	−2.213333967	Cell wall and cell processes
Rv0283 (*eccB3*)	Down	−1.489782204	Cell wall and cell processes
Rv1620c (*cydC*)	Down	−3.302613118	Intermediary metabolism and respiration
Rv1886c (*fbpB*)	Down	−1.923219182	Lipid metabolism
Rv0281	Down	−2.01561594	Lipid metabolism
Rv3545c (*cyp125*)	Up	2.103879342	Intermediary metabolism and respiration
Rv1203c	Down	−7.198592765	Conserved hypotheticals
Rv2989	Down	−3.20461473	Regulatory proteins
Rv0991c	Down	−7.786451806	Conserved hypotheticals
Rv1928c	Up	4.669685894	Intermediary metabolism and respiration
Rv0878c (PPE13)	Up	2.415321478	PE/PPE
Rv2629	Up	0.960349298	Conserved hypotheticals
Rv1205	Down	−7.651398542	Conserved hypotheticals
Rv2219A	Up	3.175822134	Cell wall and cell processes
Rv2195 (*qcrA*)	Down	−1.336408897	Intermediary metabolism and respiration
Rv0286 (PPE4)	Down	−1.456545343	PE/PPE
Rv1279	Up	2.363244433	Intermediary metabolism and respiration
Rv2297	Up	6.906052701	Conserved hypotheticals
Rv2627c	Up	1.449808908	Conserved hypotheticals
Rv3552	Up	2.886324732	Intermediary metabolism and respiration
Rv1154c	Down	−6.416507102	Conserved hypotheticals
Rv1627c	Up	1.559464153	Lipid metabolism
Rv1129c	Up	3.923881355	Regulatory proteins
Rv0289 (*espG3*)	Down	−1.541863924	Cell wall and cell processes
Rv3078 (*hab*)	Up	7.058740267	Intermediary metabolism and respiration
Rv1505c	Up	8.135570656	Conserved hypotheticals
Rv0722 (*rpmD*)	Up	5.031834752	Information pathways
Rv0129c (*fbpC*)	Up	1.398249199	Lipid metabolism
Rv1909c (*furA*)	Down	−3.361374217	Regulatory proteins
Rv1183 (*mmpL10*)	Up	1.272401462	Cell wall and cell processes
Rv0339c	Up	4.26743004	Regulatory proteins
Rv0334 (*rmlA*)	Up	3.877274953	Intermediary metabolism and respiration
Rv3226c	Up	5.924572815	Conserved hypotheticals
Rv3554 (*fdxB*)	Up	2.655054967	Intermediary metabolism and respiration
Rv1412 (*ribC*)	Down	−5.752670889	Intermediary metabolism and respiration
Rv0885	Up	2.688429483	Conserved hypotheticals
Rv2633c	Down	−1.371662275	Conserved hypotheticals
Rv3739c (PPE67)	Up	3.972189009	PE/PPE
Rv1052	Down	−6.079683034	Conserved hypotheticals
Rv2679 (*echA15*)	Up	6.474913551	Lipid metabolism
Rv1996	Up	0.766746134	Virulence, detoxification, adaptation
Rv2032 (*acg*)	Up	1.102006514	Conserved hypotheticals
Rv1846c (*blaI*)	Down	−1.679048609	Regulatory proteins
Rv3540c (*ltp2*)	Up	4.699170107	Lipid metabolism
Rv2944	Up	7.224902316	Insertion sequences and phages
Rv2280	Down	−1.619272741	Intermediary metabolism and respiration
Rv1499	Up	6.653957073	Conserved hypotheticals
Rv2857c	Up	4.523021237	Intermediary metabolism and respiration
Rv2549c (*vapC20*)	Down	−6.254071649	Virulence, detoxification, adaptation
Rv1309 (*atpG*)	Down	−1.054498325	Intermediary metabolism and respiration
Rv0847 (*lpqS*)	Up	4.7025265	Cell wall and cell processes

**TABLE 2:** Differentially expressed genes of H37Rv cholesterol versus *ΔvapC12* cholesterol

Gene ID	Log_2_-fold change	Up or down	Functional category
Rv1721c	−3.809774762	Down	Virulence, detoxification, and adaptation
Rv2378c	−7.834381397	Down	Lipid metabolism
Rv1441c	−7.414059049	Down	PE-PPE
Rv1740	−6.647609595	Down	Virulence, detoxification, and adaptation
Rv2859c	−5.030327611	Down	Intermediary metabolism and respiration

10.1128/mSystems.00855-20.1 FIG S1 (A) Growth curve of H37Rv in minimal media only, minimal media plus glycerol-only, and minimal media plus cholesterol-only media. A log-phase culture of H37Rv was washed with PBS-tyloxapol and inoculated in respective media at an absorbance of 0.005. Bacterial enumeration was done by CFU plating the cultures at regular time points. The experiment was repeated three times, and the data plotted represent the means ± the SEM. Data were analyzed using unpaired Student *t* test. (B) The growth curve of M. bovis BCG in a minimal media plus 0.1% glycerol only and minimal media plus 0.01% cholesterol only. Log-phase cultures of M. bovis BCG grown in 7H9 media enriched with OADC were washed with PBS-tyloxapol and resuspended in respective media at an absorbance of 0.005. Growth was estimated by CFU plating on 7H11+OADC plates at different time points postinoculation. Experiments were performed in triplicates, and data represent the means ± the SEM. (C) Resazurin-based estimation of the metabolic activity of M. bovis BCG grown in minimal media supplemented with minimal media plus glycerol and minimal media plus cholesterol. The culture was serially diluted in a 96-well plate in respective media. The experiment was performed in two independent sets, and the plate was incubated at 37°C for 5 days. One set of the experiment was used for recording fluorescence after adding the PrestoBlue reagent at 570 or 585 nm, whereas the other set was used for the enumeration of bacteria present in each well. The metabolic activity calculated for each well is representative of the mean fluorescent readout per bacteria from three independent experiments. Data were analyzed using an unpaired Student *t* test. *, *P* < 0.05; **, *P* < 0.01. Download FIG S1, TIF file, 0.1 MB. Copyright © 2020 Talwar et al. 2020 Talwar et al. This content is distributed under the terms of the Creative Commons Attribution 4.0 International license.

10.1128/mSystems.00855-20.2 FIG S2 (A) TraCS data representing transposon mutants of the *vapC* genes that were overrepresented by >2-fold in a cholesterol-rich media compared to a glycerol-rich media, as calculated by the number of reads detected per TA insertion site (33). (B) Log-phase cultures of H37Rv and ΔRv0665 (*ΔvapC8*) grown in the 7H9 enriched media were washed with PBS-tyloxapol and resuspended in minimal media plus 0.01% cholesterol at an absorbance of 0.005. The percent survival of *ΔvapC8* relative to wild-type H37Rv was estimated by plating the culture at day 0 and day 8 postinoculation. Download FIG S2, TIF file, 0.2 MB. Copyright © 2020 Talwar et al. 2020 Talwar et al. This content is distributed under the terms of the Creative Commons Attribution 4.0 International license.

10.1128/mSystems.00855-20.3 FIG S3 (A) Kill curve of M. bovis BCG, BCGΔ*vapC12*, and BCGΔ*vapC12:vapBC12* strains grown in glycerol media. Log-phase cultures of strains were washed with PBS-tyloxapol and inoculated at an absorbance of 0.05. The cultures were allowed to grow for 4 days before being treated with 5× MIC of rifamycin. Bacterial enumeration was performed by plating cultures on 7H11+OADC plates at various time points. The kill curve was plotted by plotting CFU. The experiment was repeated three times, and the data plotted represent the means ± the SEM. Data were analyzed using unpaired Student *t* test. (B) Volcano plot of differentially expressed genes in H37Rv grown in the cholesterol-rich media relative to the glycerol media. The transcriptome of *M. tuberculosis* exhibited 39 downregulated and 45 upregulated genes in the cholesterol-rich media relative to the glycerol media. Download FIG S3, TIF file, 0.2 MB. Copyright © 2020 Talwar et al. 2020 Talwar et al. This content is distributed under the terms of the Creative Commons Attribution 4.0 International license.

### VapC12 RNase toxin targeting proT is essential for cholesterol-mediated growth regulation in *M. tuberculosis*.

**FIG 2:**
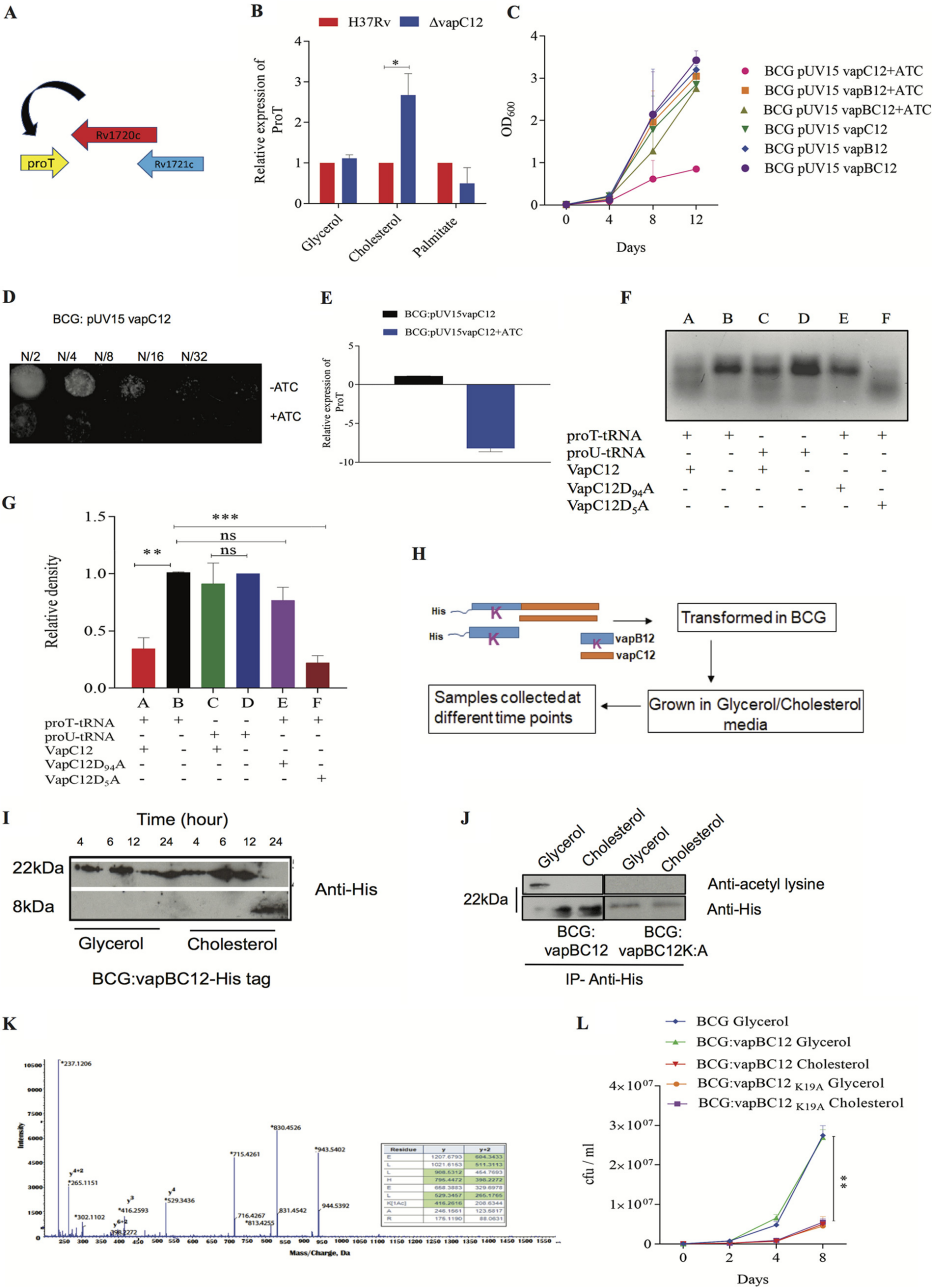
VapC12 RNase toxin targeting proT is essential for cholesterol-mediated growth regulation in *M. tuberculosis*. (A) Diagram of toxin-antitoxin *vapBC12* locus. (B) Relative expression of the proT tRNA through qRT-PCR in the *vapC12* mutant relative to the wild-type H37Rv strain grown in media containing glycerol, cholesterol, and palmitate as the sole carbon source. (C) Growth curve of M. bovis BCG strains expressing *vapC12*, *vapB12*, and *vapBC12* in the pUV15-tetO expression system under the tet-inducible promoter in 7H9+OADC media. Anhydrotetracycline (ATc), an inducer of the tet operon, was used at a concentration of 100 ng/ml and replenished every fourth day. (D) Twofold serial dilutions (N/2, N/4, N/8, N/16, and N/32) of the log-phase growing culture BCG:pUV15 *tetO:vapC12* strain grown in 7H9 broth were spotted on 7H11 agar plates with or without ATc. (E) Relative quantification of the transcript levels of the proT gene *in* BCG:pUV15*-tetO:vapC12* grown in 7H9 media with or without ATc by qRT-PCR. (F) RNase activity of purified wild-type and mutant VapC12 toxins against *in vitro*-transcribed tRNA substrates. Different wells of the gel denote different combinations of tRNA transcript and purified proteins, *viz*., wild-type VapC12 toxin protein incubated with proT (A), proT tRNA only with no protein (B), wild-type VapC12 toxin protein incubated with proU (C), proU tRNA only (D), mutant VapC12D_94_A toxin protein incubated with proT (E), and mutant VapC12D_5_A toxin protein incubated with proT (F). Each reaction mixture was incubated at 37°C for 3 h. The products of each of the reactions were run on a 3% agarose gel and visualized by adding ethidium bromide, followed by exposure to UV light. (G) Relative density of marked RNA bands in panel F quantified using ImageJ. The experiment (see panel F) was repeated three times, and data were plotted to represent the means ± the SEM. (H) Schematic representation of the protocol for the experiment to demonstrate cholesterol-specific dissociation of the antitoxin. (I) Western blot for cholesterol-specific dissociation and degradation of the antitoxin from the toxin-antitoxin complex. The His-tagged antitoxin was tracked using an anti-His antibody in the cell lysate of BCG overexpressing His-tagged antitoxin as a part of the toxin-antitoxin complex. Cell lysates were prepared by sampling cultures grown in both glycerol and cholesterol media at different time points and probed with an anti-His antibody. (J) Western blot of the protein lysates prepared from BCG overexpressing the toxin-antitoxin locus (VapBC12) with His-tagged antitoxin VapB12 and BCG strain with N-terminal His tagged VapBC12, wherein the lysine residue of the antitoxin is converted to alanine (VapB_K:A_C12). Immunoprecipitation was performed with mouse anti-His antibody and probed with rabbit anti-acetyl lysine and anti-His antibodies. To normalize for the amount of the protein, a 3-fold higher concentration of protein was loaded in the cholesterol-grown BCG sample. (K) Mass spectrometry analysis of His-tagged antitoxin protein isolated from BCG overexpressing VapBC12 complex grown in glycerol and cholesterol media. Tryptic digests of immunoprecipitated samples from glycerol- and cholesterol-grown cultures were analyzed by LC-MS/MS (Sciex, 5600 Triple-TOF). A representative MS/MS spectrum of the peptide from the glycerol-grown sample, ELLHELK(Ac)AR, was acetylated and displays a mass shift corresponding to acetylation (*m/z* 416.26) compared to the unmodified peptide from the cholesterol-grown sample. (L) Growth curve of M. bovis BCG, BCG overexpressing toxin-antitoxin (*vapBC12*), and lysine mutant (*vapB_K:A_C12*) strains in a minimal media plus 0.1% glycerol and minimal media plus 0.01% cholesterol. Bacterial enumeration was performed by CFU plating on 7H11+OADC plates at regular intervals. The experiment was performed in triplicates, and the data plotted represent means ± the SEM. Data were analyzed by using an unpaired Student *t* test. *, *P* < 0.05; **, *P* < 0.01.

Since the VapC family of toxins targets RNAs (40–42), the presence of proT-tRNA (proT) gene upstream to the *vapC12* gene was intriguing. Thus, we hypothesized that this proT tRNA can be one of the substrates for the toxin (Fig. 2A). As predicted, we observed a cholesterol-specific decrease in the proT transcript levels in wild-type strain (see Fig. S4A), and this cholesterol-specific decrease was not observed in a *vapC12-*null strain (Fig. 2B), suggesting that proT tRNA could be one of the major substrates of the VapC12 RNase toxin. To further confirm the proT specificity, we quantified the transcript levels of 10 different tRNAs that had GC-rich anticodon sequences. Surprisingly, we found no cholesterol specific differences in the abundance of any of the tested tRNAs, emphasizing that proT is one of the major substrates of the VapC12 RNase toxin (Fig. 2B; see also Fig. S4B). To further rule out the role of low growth rate contributing to the above phenotype, we quantified the relative proT expression in a WT strain (M. bovis BCG) grown in palmitate as the sole carbon source (see Fig. S5A), and we did not observe any palmitate-specific changes in the proT transcript levels (Fig. 2B).

10.1128/mSystems.00855-20.4 FIG S4 (A) Relative expression of the proT tRNA through qRT-PCR in wild-type H37Rv strain grown in media containing glycerol and cholesterol as the sole carbon source. (B) Relative expression of 10 tRNAs through qRT-PCR in wild-type H37Rv and *ΔvapC12* grown in the cholesterol-rich media. Download FIG S4, TIF file, 0.1 MB. Copyright © 2020 Talwar et al. 2020 Talwar et al. This content is distributed under the terms of the Creative Commons Attribution 4.0 International license.

10.1128/mSystems.00855-20.5 FIG S5 (A) Growth curve analysis of BCG grown in minimal media supplemented with 0.1% glycerol and 50 mg/ml palmitate. Log-phase cultures of wild-type BCG grown in 7H9 media enriched with OADC was washed with PBS-tyloxapol and resuspended in respective media at an absorbance of 0.005. Growth was estimated by CFU plating on 7H11+OADC plates at different time points postinoculation, and colonies were counted after 3 weeks of incubation of plates at 37°C. Experiments were performed in triplicates, and data represent the means ± the SEM. (B) Twofold serial dilutions (N/2, N/4, N/8, N/16, N/32, and N/64) of the log-phase growing culture BCG:pUV15 *tetO:vapC12* strain grown in 7H9 broth and minimal media plus cholesterol were spotted onto 7H11 agar plates with or without ATc. Download FIG S5, TIF file, 0.2 MB. Copyright © 2020 Talwar et al. 2020 Talwar et al. This content is distributed under the terms of the Creative Commons Attribution 4.0 International license.

In contrast to the findings of previous studies (27, 43), we observed that overexpression of the putative *vapC12* toxin gene in a WT M. bovis BCG background reduced the proT transcript levels eliciting significant growth defect (Fig. 2C to E). This phenotype was more pronounced in the presence of cholesterol (see Fig. S5B). Furthermore, the toxin phenotype was reversed if the *vapC12* toxin gene was coexpressed along with its cognate *vapB12* antitoxin gene (Fig. 2C) (44). To further validate whether proT is the substrate of VapC12 toxin, we generated recombinant His-tagged VapC12 toxin expressed and purified in a heterologous E. coli expression system (see Fig. S6A). When exposed to *in vitro* transcribed tRNAs, namely, proT and proU, the purified recombinant toxin specifically cleaved proT (Fig. 2F and G). Furthermore, we mutated two highly conserved aspartate residues D_5_ and D_94_ in the PIN domain of the toxin to alanine (see Fig. S6B). An aspartate (D)-to-alanine (A) conversion of the 94th residue of the VapC12 toxin failed to cleave the substrate. This inactivation of the toxin by D_94_A substitution may be due to its inability to bind to Mg^2+^, which is a critical cofactor required for its activity (40, 45). Although we used proU-tRNA (proU) as our control, we do not rule out the possibility of other tRNAs being a VapC12 substrate. A D_5_A substitution did not affect the RNase activity of the toxin.

10.1128/mSystems.00855-20.6 FIG S6 (A) Purified recombinant VapC12, VapC12 D_5_A, and VapC12 D_94_A proteins were subjected to SDS-PAGE and probed with an anti-His antibody. (B) Multiple sequence alignment of VapC toxins indicating conserved aspartate residues in the PIN domain of toxins. Download FIG S6, TIF file, 0.2 MB. Copyright © 2020 Talwar et al. 2020 Talwar et al. This content is distributed under the terms of the Creative Commons Attribution 4.0 International license.

Activation of the type II toxins is primarily driven by the degradation of the corresponding antitoxin. Therefore, to study cholesterol-specific degradation of the antitoxin, we generated a recombinant M. bovis BCG overexpressing both toxin and antitoxin in which the antitoxin was tagged with 6×His at the N terminus. By Western blot analysis, we observed that in the presence of cholesterol there was a time-dependent dissociation and degradation of the antitoxin (VapB12) protein that possibly resulted in the generation of the active monomeric form of the cognate toxin (Fig. 2H and I). Surprisingly, the only lysine residue, K_19_, of the antitoxin lost its acetylation in the presence of cholesterol (Fig. 2J), and this we believe could possibly be a signal for cholesterol-induced degradation of antitoxin and the subsequent activation of VapC12 toxin. We validated this by liquid chromatography-tandem mass spectrometry (LC-MS/MS) wherein we observed that, unlike cholesterol, acetylation of the lysine residue (K_19_) of the antitoxin protein was observed only in the protein lysates generated from glycerol-grown *M. tuberculosis* culture (Fig. 2K). Surprisingly, the protein coverage of the antitoxin peptides isolated from *M. tuberculosis* grown in cholesterol was ≥95% except for the peptide LHELK with a sequence coverage of ≥50 < 95 (see Fig. S7 in the supplemental material). This could be attributed to a cholesterol-specific degradation of the antitoxin (Fig. 2I). To confirm this further, we generated a recombinant M. bovis BCG strain overexpressing the antitoxin protein harboring a lysine to alanine (K_19_A) substitution and, as expected due to the absence of the lysine residue, the antitoxin could not be acetylated (Fig. 2J). This resulted in constitutive degradation of the antitoxin leading to growth inhibition, independent of the carbon source, in mycobacteria (Fig. 2L).

10.1128/mSystems.00855-20.7 FIG S7 Protein sequence coverage of peptides from BCG:*vapBC12* His-tagged overexpression strain grown in glycerol and cholesterol media, where gray indicates no match or 0 peptide confidence, red indicates >0 and <50 peptide confidence, yellow indicates ≥50 and <95 peptide confidence, and green indicates ≥95 peptide confidence. Download FIG S7, TIF file, 0.1 MB. Copyright © 2020 Talwar et al. 2020 Talwar et al. This content is distributed under the terms of the Creative Commons Attribution 4.0 International license.

### Cholesterol-dependent activation of *vapC12* toxin generates and enriches the slow-growing population in the *M. tuberculosis* culture.

**FIG 3:**
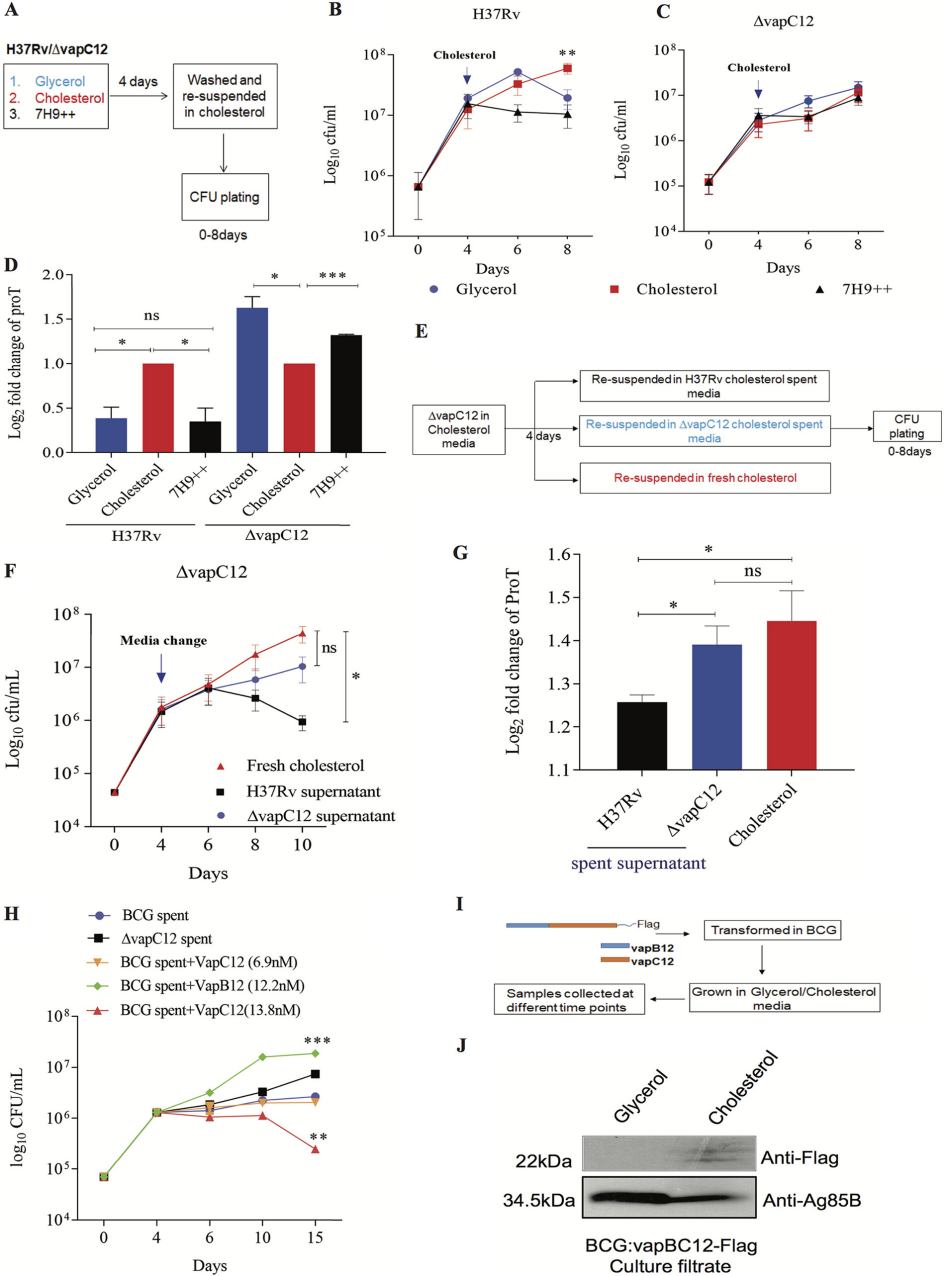
Cholesterol-dependent activation of *vapC12* toxin generates and enriches the persister population in the *M. tuberculosis* culture. (A) Schematic representation of the persister enrichment experiment. (B and C) Growth curve of H37Rv and *vapC12* mutant strains grown in 7H9-enriched, 0.1% glycerol, and 0.01% cholesterol media for the first 4 days and then resuspended in a cholesterol-rich media for subsequent days. Bacterial enumeration was performed by plating cultures on 7H11+OADC plates at various time points. The experiment was performed in triplicates, and the data plotted represent the means ± the SEM. Data were analyzed using unpaired Student *t* test. *, *P* < 0.05. (D) Expression analysis of proT tRNA through qRT-PCR in H37Rv and *vapC12* mutant strains at day 8 relative to day 4 of the persister enrichment growth curve (see panels B and C). (E) Schematic representation of growth curves obtained from spent media from wild-type H37Rv and *vapC12* mutant strains grown in cholesterol. (F) The *vapC12* mutant strain was grown in a medium containing 0.01% cholesterol in triplicate for the first 4 days and then resuspended in spent medium from H37Rv, *vapC12* mutant, and fresh cholesterol individually. Bacterial enumeration was performed by plating cultures on 7H11+OADC plates. The experiment was repeated three times, and the data plotted represent the means ± the SEM. Data were analyzed using unpaired Student *t* test. *, *P* < 0.05; **, *P* < 0.01. (G) Expression analysis of proT tRNA through qRT-PCR in the *vapC12* mutant strain grown in a cholesterol-spent media at day 10 of the growth curve relative to the culture grown in a fresh cholesterol-rich media at day 4 (see panel F). (H) The BCG *vapC12* mutant strain was grown in a media containing 0.01% cholesterol in triplicate for the first 4 days and then resuspended in spent media from wild-type BCG, *vapC12* mutant, and wild-type BCG supplemented with purified VapB12 antitoxin (12.2 nM) and VapC12 toxin at two different concentrations (6.9 and 13.8 nM). Bacterial enumeration was performed by CFU plating on 7H11+OADC plates. The experiment was repeated three times, and the data plotted represent the means ± the SEM. Data were analyzed using unpaired Student *t* test. *, *P* < 0.05 **, *P* < 0.01. (I) Schematic representation of the experiment to demonstrate that toxin is secreted out in the culture filtrate of BCG. (J) Western blot showing the Flag-tagged toxin in the culture filtrate of BCG strain overexpressing the toxin-antitoxin complex (VapBC12). The culture filtrate was probed with an anti-Flag antibody to detect the toxin protein, the anti-Ag85B antibody was used as a positive control for the secretory protein, and an anti-GroEL1 antibody was used as a negative control to ensure no lysis of bacterial cells occurred during sample preparation.

To further evaluate cholesterol-induced activation of VapC12 toxin and the subsequent slowdown of *M. tuberculosis* growth, a log-phase culture of WT *M. tuberculosis* grown in an enriched (7H9+OADC [oleic acid-albumin-dextrose-catalase]), cholesterol, and glycerol medium was subsequently exposed to cholesterol, and the effect on bacterial growth was assessed (Fig. 3A). Interestingly, while there was no change in the growth rate of culture previously exposed to cholesterol, a significant reduction in the growth of *M. tuberculosis* was observed in cultures that were growing in both glycerol and the enriched media before the cholesterol switch (Fig. 3B). This phenotype was again found to be dependent on the presence of the *vapC12* toxin gene (Fig. 3C). These results indicate a *vapC12*-mediated cholesterol-dependent reduction in *M. tuberculosis* growth. Compared to the glycerol media, the reduction in the growth rate was more prominent in the enriched media. We also found a reduction in the transcript levels of the proT tRNA in cholesterol-exposed *M. tuberculosis* culture previously grown in either enriched media or glycerol media (Fig. 3D), further confirming the finding that the difference in growth is due to toxin-mediated degradation of proline-tRNA. To explore the extracellular role of VapC12 toxin in restricting the growth of fast-growing bacteria in a heterogeneous population (46, 47), we suspended a log-phase culture of the *M. tuberculosis vapC12* mutant strain separately in the spent media harvested from either the cholesterol-grown WT or *vapC12* mutant strain (Fig. 3E). A decrease in the *vapC12* mutant CFU was observed only in culture exposed to supernatant isolated from the cholesterol-grown WT strain, suggesting that either VapC12 toxin directly or a VapC12-dependent secretory protein selectively enriches the slow-growing persister population in *M. tuberculosis* cultures in a cholesterol-rich environment (Fig. 3F). The quantification of proT levels in these cultures further suggested that the observed phenotype was indeed due to differences in the toxin-mediated proT cleavage (Fig. 3G). Furthermore, neutralization of the toxin in WT spent media by adding a purified antitoxin increased the *vapC12* mutant CFU; similarly, the addition of the purified toxin in spent media from *ΔvapC12* resulted in a dose-dependent decrease in the CFU of the *vapC12*-null strain (Fig. 3H). Finally, we demonstrated that VapC12 toxin was detected only in the culture filtrate isolated from cholesterol-grown M. bovis BCG overexpressing Flag-tagged VapC12 and not from the glycerol-grown culture (Fig. 3I and J).

### Significance of *vapC12*-mediated downregulation of the proT-codon enriched proteins.

**FIG 4:**
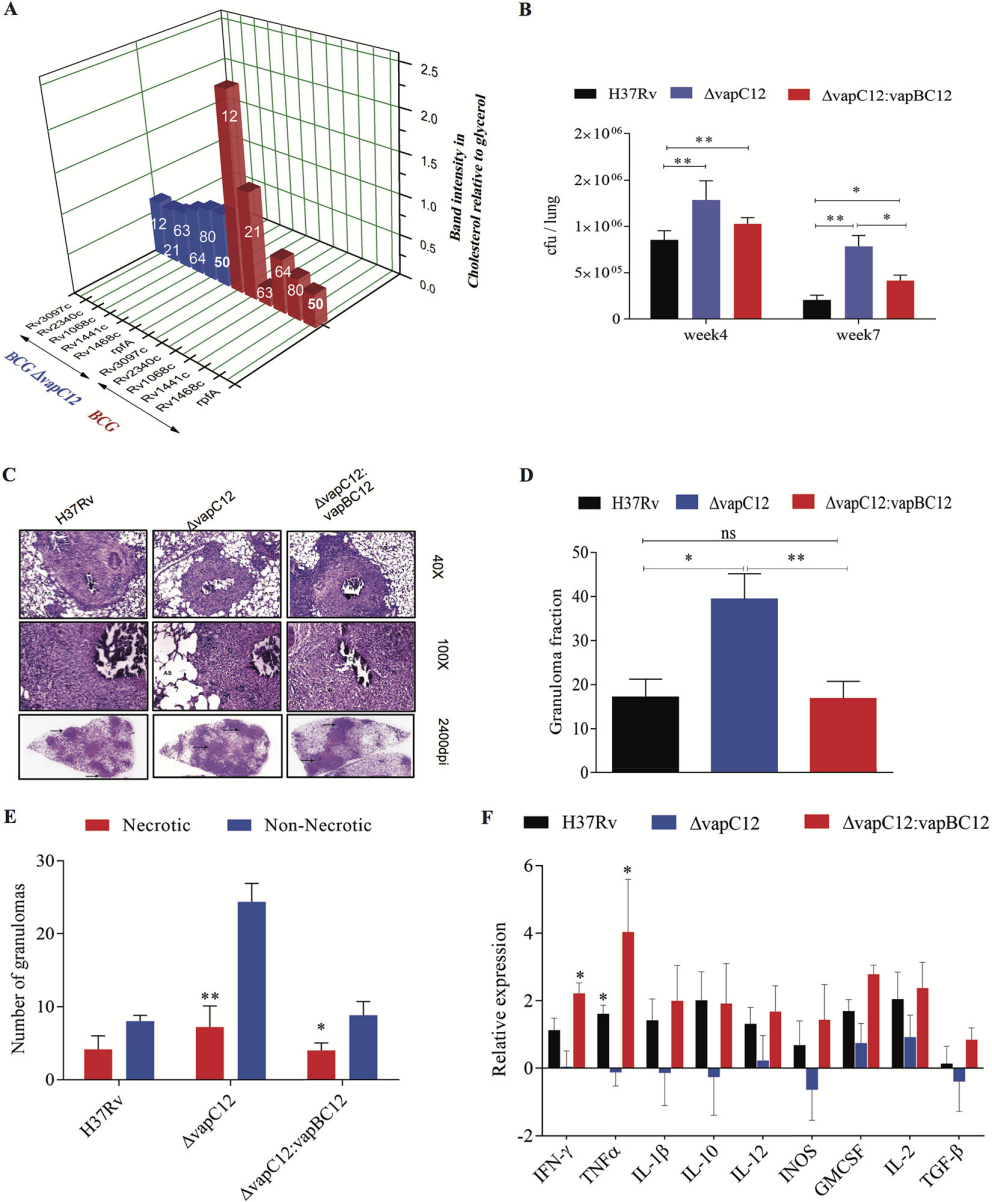
*vapC12*-mediated downregulation of the proT-encoded proline-rich proteins is essential for the persistence of *M. tuberculosis* in a guinea pig model of infection. (A) Relative band intensity representing the expression of His-tagged PE-PGRS and RpfA proteins in BCG and *vapC12* mutant strains grown in glycerol and cholesterol media. The numbers on each individual bar represent the percentages of ProT codon in that particular protein. The protein lysates were prepared from overexpressed strains with an OD of 0.8 to 1. The samples were run on SDS-PAGE and probed with an anti-His antibody. (B) Bacterial load in the lungs of guinea pigs infected with H37Rv, *ΔvapC12*, and *ΔvapC12*:*vapBC12 M. tuberculosis* strains. At the designated time points, the lungs were homogenized in 4 ml of saline, and 10-fold serial dilutions of homogenates were plated on 7H11+OADC plates. Each group constituted six guinea pigs per time point. The data plotted represent the means ± the SEM. Significant differences observed between groups are indicated. Data were analyzed using the Mann-Whitney U test (**, *P* < 0.01; *, *P* < 0.05). (C) Photomicrographs of H&E-stained (40× and 100×) and high-resolution scanning (2,400 dpi) of lung sections from guinea pigs infected with different strains of *M. tuberculosis* at 7 weeks postinfection. (D) Granuloma fraction of the lung tissue samples of guinea pigs infected with different strains of *M. tuberculosis*, based on the semiquantitative estimation of the fraction of the lung tissue covered with granuloma. Data were analyzed using the Mann-Whitney U test (*, *P* < 0.05; **, *P* < 0.01). (E) Total number of necrotic and nonnecrotic granulomas in the lung tissue samples of guinea pigs infected with different strains of *M. tuberculosis*. Data were analyzed using the Mann-Whitney U test (*, *P* < 0.05; **, *P* < 0.01; ***, *P* < 0.001). (F) Cytokine profiling of animals infected with H37Rv, *ΔvapC12*, and *ΔvapC12*:*vapBC12* strains of *M. tuberculosis*. RNA was extracted from the spleens of infected animals at 7 weeks postinfection. The relative expression of cytokines in different groups of animals was quantified through qRT-PCR. Data were normalized with the findings of the uninfected group. The data plotted represent the means ± the SEM. Data were analyzed using the Mann-Whitney test (*, *P* < 0.05; **, *P* < 0.01).

To investigate the implications of proT degradation in cholesterol-mediated growth modulation, a genomewide *in silico* analysis was performed to determine the frequency of the proT tRNA codon in each *M. tuberculosis* gene (see Fig. S8A in the supplemental material). The mycobacterial genome has four designated proline tRNAs (pro-T, pro-Y, pro-U, and pro-X) that incorporate the proline residue to a nascent polypeptide during translation. The results of the *in silico* analysis revealed that pro-T and pro-Y encode 85.53% of the total proline incorporated into the *M. tuberculosis* H37Rv proteome, with pro-Y (CCG) and pro-T (CCC) codon usage being 63.6 and 36.4%, respectively (see Fig. S8A). A list of 136 *M. tuberculosis* genes that had at least 60% of the proline encoded by proT tRNA was identified (Table 3). In contrast, a functional categorization revealed that proline incorporated in the PE-PGRS protein family has a significantly higher percentage (56.2%) of proline codons that require the proT tRNA. Besides, a gradient in the percentage of the proT codon usage was observed (see Fig. S8B). This led to speculation that the expression of antigenic proteins belonging to the PE-PGRS family, which contain various numbers and frequencies of the proT codons, are differentially regulated in a cholesterol-rich environment. We hypothesized that through VapC12 toxin-mediated degradation of proT in cholesterol, *M. tuberculosis* downregulates the expression of these antigenic proT-rich PE-PGRS proteins. We selected a set of five different PE-PGRS proteins with varying proT codon usage and found that the expression levels of PE-PGRS proteins in wild-type M. bovis BCG grown in cholesterol media were inversely correlated with the frequency of proT encoded proline in the gene (Fig. 4A; see Fig. S8C). This phenotype was completely dependent on the presence of VapC12 toxin because no medium-specific differences were observed in the expression of the aforementioned proteins in the *vapC12*-null strain (Fig. 4A; see also Fig. S8C). We also checked the expression of these genes at the transcript level and, to our surprise, we found that, in comparison to glycerol, all other genes (except for *Rv1068c*) were found to be transcriptionally upregulated in cholesterol. These findings further confirm that the cholesterol-dependent decrease in the expression of these proteins is perhaps regulated at the translation level (see Fig. S8D). The factor *rpfA*, one of the four resuscitation-promoting factors (*rpfA* to *rpfD*) of *M. tuberculosis*, has 53.16% of its proline encoded by proT. These Rpf proteins are peptidoglycan glycosidases required for the activation of quiescent bacteria; this is an essential step for the reactivation of *M. tuberculosis* in an *in vitro* model (48). In addition, *rpfA* in *M. tuberculosis* has been reported to be secretory through a *sec*-dependent pathway and speculated to be involved in modulating host during the reactivation process (49). Interestingly, in comparison to glycerol, we found a *vapC12*-dependent decrease in the expression of RpfA protein in cholesterol grown M. bovis BCG strain overexpressing a 6×His-tagged *M. tuberculosis* RpfA protein. We believe that the cholesterol-dependent regulation of RpfA levels by the VapC12 toxin is a mechanism by which *M. tuberculosis* sustains latency during infection.

**TABLE 3:** Functional categorization of genes with proT codon usage above 60%

Gene ID	Functional category	% proT
Rv0596c	Virulence, detoxification, adaptation	60
Rv1952	Virulence, detoxification, adaptation	60
Rv2865	Virulence, detoxification, adaptation	60
Rv2549c	Virulence, detoxification, adaptation	66.66666667
Rv1956	Virulence, detoxification, adaptation	71.42857143
Rv1982A	Virulence, detoxification, adaptation	75
Rv2863	Virulence, detoxification, adaptation	80
Rv0300	Virulence, detoxification, adaptation	100
Rv0550c	Virulence, detoxification, adaptation	100
Rv1103c	Virulence, detoxification, adaptation	100
Rv2760c	Virulence, detoxification, adaptation	100
Rv0737	Regulatory proteins	60
Rv3557c	Regulatory proteins	60
Rv3050c	Regulatory proteins	62.5
Rv1994c	Regulatory proteins	66.66666667
Rv0348	Regulatory proteins	71.42857143
Rv2021c	Regulatory proteins	100
Rv3653	PE/PPE	60
Rv0872c	PE/PPE	61.11111111
Rv2853	PE/PPE	61.11111111
Rv0354c	PE/PPE	62.5
Rv2162c	PE/PPE	62.5
Rv1068c	PE/PPE	63.63636364
Rv3512	PE/PPE	64.28571429
Rv0833	PE/PPE	64.70588235
Rv1441c	PE/PPE	64.70588235
Rv3367	PE/PPE	64.70588235
Rv1452c	PE/PPE	65.2173913
Rv1169c	PE/PPE	66.66666667
Rv1840c	PE/PPE	66.66666667
Rv0578c	PE/PPE	68.29268293
Rv0747	PE/PPE	68.42105263
Rv1468c	PE/PPE	80
Rv3508	PE/PPE	85.18518519
Rv3514	PE/PPE	90.32258065
Rv0470c	Lipid metabolism	60
Rv0972c	Lipid metabolism	60
Rv2724c	Lipid metabolism	60
Rv2982c	Lipid metabolism	60
Rv3221c	Lipid metabolism	100
Rv0771	Intermediary metabolism and respiration	60
Rv1851	Intermediary metabolism and respiration	60
Rv1826	Intermediary metabolism and respiration	60
Rv2499c	Intermediary metabolism and respiration	60
Rv2511	Intermediary metabolism and respiration	60
Rv2250A	Intermediary metabolism and respiration	63.63636364
Rv1692	Intermediary metabolism and respiration	64.70588235
Rv1311	Intermediary metabolism and respiration	66.66666667
Rv1555	Intermediary metabolism and respiration	66.66666667
Rv1990A	Intermediary metabolism and respiration	66.66666667
Rv2539c	Intermediary metabolism and respiration	66.66666667
Rv3145	Intermediary metabolism and respiration	66.66666667
Rv2421c	Intermediary metabolism and respiration	70
Rv3624c	Intermediary metabolism and respiration	70
Rv2754c	Intermediary metabolism and respiration	71.42857143
Rv0558	Intermediary metabolism and respiration	72.72727273
Rv0814c	Intermediary metabolism and respiration	75
Rv1305	Intermediary metabolism and respiration	75
Rv3118	Intermediary metabolism and respiration	75
Rv3154	Intermediary metabolism and respiration	77.77777778
Rv2537c	Intermediary metabolism and respiration	80
Rv0137c	Intermediary metabolism and respiration	81.81818182
Rv0763c	Intermediary metabolism and respiration	83.33333333
Rv0741	Insertion sequences and phages	60
Rv1586c	Insertion sequences and phages	60
Rv2014	Insertion sequences and phages	71.42857143
Rv3638	Insertion sequences and phages	71.42857143
Rv1702c	Insertion sequences and phages	74.19354839
Rv1584c	Insertion sequences and phages	75
Rv0094c	Insertion sequences and phages	77.27272727
Rv3467	Insertion sequences and phages	77.27272727
Rv1765A	Insertion sequences and phages	100
Rv1316c	Information pathways	60
Rv2069	Information pathways	60
Rv2906c	Information pathways	60
Rv2058c	Information pathways	66.66666667
Rv2056c	Information pathways	66.66666667
Rv1643	Information pathways	66.66666667
Rv0722	Information pathways	100
Rv2441c	Information pathways	100
Rv3053c	Information pathways	100
Rv3462c	Information pathways	100
Rv1772	Conserved hypotheticals	60
Rv0678	Conserved hypotheticals	60
Rv0607	Conserved hypotheticals	60
Rv3678A	Conserved hypotheticals	60
Rv1590	Conserved hypotheticals	60
Rv2283	Conserved hypotheticals	60
Rv2426c	Conserved hypotheticals	60
Rv2438A	Conserved hypotheticals	60
Rv2558	Conserved hypotheticals	60
Rv3224B	Conserved hypotheticals	60
Rv0323c	Conserved hypotheticals	63.63636364
Rv2257c	Conserved hypotheticals	64.70588235
Rv0078B	Conserved hypotheticals	66.66666667
Rv0181c	Conserved hypotheticals	66.66666667
Rv0530A	Conserved hypotheticals	66.66666667
Rv1120c	Conserved hypotheticals	66.66666667
Rv2820c	Conserved hypotheticals	66.66666667
Rv2239c	Conserved hypotheticals	66.66666667
Rv2342	Conserved hypotheticals	66.66666667
Rv2923c	Conserved hypotheticals	66.66666667
Rv3472	Conserved hypotheticals	66.66666667
Rv3033	Conserved hypotheticals	71.42857143
Rv0028	Conserved hypotheticals	75
Rv1890c	Conserved hypotheticals	75
Rv2603c	Conserved hypotheticals	75
Rv1066	Conserved hypotheticals	80
Rv2049c	Conserved hypotheticals	100
Rv0378	Conserved hypotheticals	100
Rv1893	Conserved hypotheticals	100
Rv1993c	Conserved hypotheticals	100
Rv2548A	Conserved hypotheticals	100
Rv2738c	Conserved hypotheticals	100
Rv3440c	Conserved hypotheticals	100
Rv0011c	Cell wall and cell processes	60
Rv0431	Cell wall and cell processes	60
Rv1463	Cell wall and cell processes	60
Rv1973	Cell wall and cell processes	60
Rv2856	Cell wall and cell processes	60
Rv2936	Cell wall and cell processes	61.53846154
Rv3864	Cell wall and cell processes	61.9047619
Rv3312A	Cell wall and cell processes	62.5
Rv0583c	Cell wall and cell processes	65
Rv2732c	Cell wall and cell processes	66.66666667
Rv3277	Cell wall and cell processes	66.66666667
Rv0476	Cell wall and cell processes	75
Rv1881c	Cell wall and cell processes	75
Rv2301	Cell wall and cell processes	75
Rv3271c	Cell wall and cell processes	75
Rv0900	Cell wall and cell processes	100
Rv0039c	Cell wall and cell processes	100
Rv0288	Cell wall and cell processes	100
Rv2520c	Cell wall and cell processes	100
Rv3789	Cell wall and cell processes	100
Rv3857c	Cell wall and cell processes	100

10.1128/mSystems.00855-20.8 FIG S8 (A) Genomewide *in silico* analysis of the codon usage of proT and proY tRNA in each gene belonging to all 10 functional groups in *M. tuberculosis*. Data for codon usage can be obtained from the link provided in Materials and Methods. (B) Codon usage of proT and proY tRNA in the PE-PGRS group of genes of *M. tuberculosis*. (C) Relative expression of His-tagged PE-PGRS and RpfA proteins in BCG and *vapC12* mutant strains grown in glycerol and cholesterol media. The protein lysates were prepared from overexpression strains with an OD of 0.8 to 1. The samples were run on SDS-PAGE and probed with an anti-His antibody. (D) Expression analysis of PE-PGRS and *rpfA* through qRT-PCR in the M. bovis BCG grown in cholesterol media relative to M. bovis BCG grown in glycerol media. Download FIG S8, TIF file, 0.4 MB. Copyright © 2020 Talwar et al. 2020 Talwar et al. This content is distributed under the terms of the Creative Commons Attribution 4.0 International license.

### The *vapC12* mutant strain of *M. tuberculosis* demonstrates a hypervirulence phenotype in the guinea pig infection model.

Before initiating any infection-related studies, we confirmed the presence of phthiocerol dimycocerosates (PDIM), a virulence-associated surface lipid, in the wild type, *vapC12* mutant, and the complemented strains (see Fig. S9A in the supplemental material). To assess the possible role of *vapC12* in the host, we first infected mouse bone marrow-derived macrophages (BMDM) with WT and *vapC12*-null strains. The null strain demonstrated increased replication in BMDM, which was abolished in the complemented strain (see Fig. S9B). Of note, the *vapC12*-null strain also showed an enhanced growth phenotype when exposed to higher oxidative and nitrosative stress conditions (see Fig. S9C). Next, we infected guinea pigs with WT, *vapC12*-null, and complemented *M. tuberculosis* strains. At 7 weeks postinfection, a higher bacterial load was observed in the lungs of guinea pigs infected with the *vapC12*-null strain compared to animals infected with a WT or complemented strain (Fig. 4B). A similar profile was observed in the spleen (see Fig. S9D). In comparison to the wild type, gross examination of the lungs infected with the mutant strain revealed higher inflammation and enhanced tissue damage. Histological examination of the lungs infected with the wild-type strain at 7 weeks postinfection revealed a typical necrotic granuloma with caseated nuclei surrounded by a dense fibrous layer (Fig. 4C and D). In contrast, at the same time postinfection, lung tissues of guinea pigs infected with the *vapC12*-null strain displayed an increased number of granulomas (Fig. 4C and D; see also Fig. S9E), which were predominantly diffused and nonnecrotic, devoid of any outer fibrous layer (Fig. 4E). We also observed that these less-defined granulomas were infiltrated with a greater admixture of cell types, including neutrophils. Surprisingly, animals infected with the *vapC12*-null strain failed to induce an inflammatory response, as indicated by a decreased mRNA expression of inflammatory cytokines (Fig. 4F). Guinea pigs infected with a complemented strain had a phenotype similar to those infected with a WT strain (Fig. 4C to F).

10.1128/mSystems.00855-20.9 FIG S9 (A) Apolar lipid extracts isolated from wild-type, *ΔvapC12*, and *ΔvapC12:vapBC12 M. tuberculosis* strains separated by TLC in petroleum ether-diethyl ether (90:10 [vol/vol]) and detected by iodine vapors showing PDIMs and TAG. (B) Relative survival of wild-type H37Rv, *ΔvapC12*, and *ΔvapC12:vapBC12* strains in mouse BMDM. Infection was performed at an MOI of 1, and CFU plating was performed at day 0 and day 7 for bacterial enumeration on 7H11+OADC plates. The experiment was repeated three times, and the data plotted represent the means ± the SEM. The data were analyzed using unpaired Student *t* test. *, *P* < 0.05; **, *P* < 0.01. (C) H37Rv and *ΔvapC12* strains were subjected to different stress conditions: nitrosative stress with 200 μM of Deta-NO for 48 h and oxidative stress with 5 mM of H_2_O_2_ treatment for 6 h. The percent survival of *ΔvapC12* relative to the wild-type strain was calculated by plating cultures at day 0 and respective time points. (D) Bacterial load in the spleen of guinea pigs infected with H37Rv, *ΔvapC12*, and *ΔvapC12:vapBC12 M. tuberculosis* strains. At the designated time points, spleens were homogenized in 4 ml of saline, and 10-fold serial dilutions of homogenates were plated on 7H11+OADC plates. Each group constituted six guinea pigs per time point. The data plotted represent the means ± the SEM. Significant differences observed between groups are indicated. The data were analyzed using the Mann-Whitney U test (**, *P* < 0.01; *, *P* < 0.05). (E) Gross pathology of the lungs and spleen of guinea pigs infected with various strains of *M. tuberculosis* at 7 weeks postinfection. Download FIG S9, TIF file, 0.4 MB. Copyright © 2020 Talwar et al. 2020 Talwar et al. This content is distributed under the terms of the Creative Commons Attribution 4.0 International license.

## DISCUSSION

Chronic infections necessitate the etiologic agent to persist inside the host for an extended duration. *M. tuberculosis* remarkably adapts to a very hostile niche by augmenting its ability to thrive inside the host for decades. The pathogen’s ability to modulate host immune response and its capacity to tolerate high concentrations of antimycobacterial drugs are key to its persistence. To do so, *M. tuberculosis* senses various stage-specific environmental cues and accordingly regulates the expression of various proteins that eventually help the pathogen to attain distinct phenotypes critical for long-term survival. Inside the host, *M. tuberculosis* encounters an extraordinary challenge of surviving on host-derived nutrients and subsequently creating a niche conducive for its growth. Conversely, the host has developed ways and means to deprive the unwanted guest of critical nutrients, including the much-needed carbon source. Although we have earlier demonstrated that the utilization of host cholesterol is essential for disease persistence in tuberculosis, the role of cholesterol utilization and the subsequent mechanism leading to this phenotype is largely not well defined.

In the present study, we demonstrated that cholesterol utilization results in an increase in the frequency of generation of antibiotic persisters in mycobacteria. This phenotype was abrogated in an *M. tuberculosis* RNase toxin *vapC12*-null strain. Mechanistically, we also identified *M. tuberculosis* proT tRNA as one of the substrates of the VapC12 RNase toxin and that the toxin-mediated modulation of the proT tRNA levels regulate antibiotic persistence in mycobacteria. Finally, using a guinea pig model of *M. tuberculosis* infection, we demonstrated that a reduction in the frequency of generation of *in vitro* antibiotic persisters significantly enhances the ability of *vapC12*-null strain to grow inside the host affecting disease persistence (50). In line with the recently established definitions and guidelines (51), our data suggest that in tuberculosis both antibiotic and disease persistence, either individually or in tandem, influence the disease progression and treatment outcomes. Coevolution for centuries has molded *M. tuberculosis* to adapt and utilize host-derived lipids, including cholesterol as a preferred carbon source (52, 53). Although *M. tuberculosis* has access to multiple carbon sources inside the host (15, 16), the utilization of host cholesterol is essential for long-term persistence. While nutrient-dependent growth modulation is very common (54, 55), our data for the first time indicate its effect on antibiotic and disease persistence during *M. tuberculosis* infection.

Interestingly, a decrease in the expression of *esx3* locus, a type VII secretion system critical (39) for iron uptake (36, 37), during cholesterol utilization suggests that *M. tuberculosis* deprives itself of iron to restrict growth under cholesterol-rich condition. We also found that the cholesterol-exposed *M. tuberculosis* downregulates the expression of genes belonging to the electron transport chain, resulting in a sharp decline in the intracellular ATP levels. Our data are in line with similar studies implicating that a lower ATP concentration leads to disease persistence in several species of bacteria, including *M. tuberculosis* (38, 39, 56, 57). These findings suggest that the modulation of intracellular ATP levels by a *vapC12* gene-encoded protein might have a role in cholesterol-specific growth modulation in *M. tuberculosis*. Obtaining mechanistic insights into pathways leading to VapC12 toxin-dependent regulation of intracellular ATP levels would be an interesting area for future research. In addition, an increase in the transcript levels of DosR regulon genes in the cholesterol media suggests that, in addition to hypoxia, sensing of intracellular cholesterol by *M. tuberculosis* can trigger the induction of DosR regulon genes in *M. tuberculosis*. Surprisingly, despite the cholesterol-specific growth differences observed between the WT and *vapC12* mutant strains, the differences observed in the transcript levels were very minimal, implicating posttranscriptional regulation for the observed phenotype. Our findings demonstrated growth modulation specifically attributed to the abundance of proline tRNA levels modulated by activation of VapC12 RNase toxin. Although studies describing tRNA-dependent growth modulation have been reported (28, 58, 59), our study describing the mechanism of nutrient-dependent regulation of tRNA abundance modulating growth is a novel finding.

Posttranslational modifications (PTMs) confer diversity to regulatory mechanisms that control various cellular pathways. Two of the most extensively studied PTMs are phosphorylation and acetylation. Together, they are known to regulate the stability and activity of proteins in both eukaryotes and prokaryotes. Lysine acetylation is known to regulate various cellular pathways conserved across species, including mycobacteria (60, 61). Our growth curve and mass spectrometry data also suggest that the cholesterol-mediated growth modulation is triggered by deacetylation of the only lysine residue present in the antitoxin. This assumes importance since *M. tuberculosis* in the host is known to persist inside foamy macrophages rich in cholesterol (18).

Our findings also suggest that the proT-encoded proline-rich proteome of *M. tuberculosis*, including PE-PGRS proteins, are essential for cholesterol-induced growth modulation, leading to antibiotic persistence. We predict that VapC12-mediated differential expression of these immunoregulatory PE-PGRS proteins critically regulates the disease progression and hence influences the treatment outcomes. Interestingly, our finding suggests that an increased exposure of *M. tuberculosis* to host cholesterol downregulates the expression of these immunomodulatory proteins, leading to enhanced proinflammatory cytokine secretion. This pathogen-driven proinflammatory response helps *M. tuberculosis* to establish a long-term niche inside the host in the form of a well-defined structure called granuloma: a hallmark of tuberculosis infection. *M. tuberculosis* also induces the infiltration of neutrophils in the lung tissues of infected mice. A recent report suggests that a subset of these tissue-infiltrated neutrophils provides a permissive intracellular niche facilitating increased survival of *M. tuberculosis* inside the host. These granulocytes are also reported to be involved in tissue damage and induce immunoregulatory functions by actively secreting anti-inflammatory cytokines (62). In comparison to the wild type, an increase in neutrophil infiltration observed in the lungs of guinea pigs infected with *vapC12* mutant strain possibly explains the enhanced pathology and increase in mutant growth fitness phenotype observed in guinea pigs. Our data possibly indicate that a *vapC12*-mediated regulation of granulocyte infiltration during infection is essential for disease persistence in tuberculosis. The role of the temporospatial expression of critical *M. tuberculosis* surface antigens in host immune response and its effect on the recruitment of immune cells to the site of infection would require a more integrative approach and in-depth immunophenotyping studies.

VapC12-mediated proT codon-based differential expression of various *M. tuberculosis* proteins, including PE-PGRS, is a novel finding. Interestingly, the presence of an unusually high number of the *vapBC* TA pairs in *M. tuberculosis*, together with the existing posttranslational modification, we believe, might add to the existing complexity of gene regulation at the posttranscriptional level. A comprehensive analysis of the stage-specific regulation of the expression of *M. tuberculosis* proteins and its implications on the disease progression will be an interesting area for future research. While pathogen rewiring their metabolic pathways for disease persistence is quite well studied (63, 64), there are fewer studies on the modulation of host immune response by the temporospatial expression of *M. tuberculosis* surface antigens contributing to disease persistence. Our study suggests that both the growth modulation and differential expression of surface antigens by *M. tuberculosis* might be a pathogen-driven mechanism that contributes to disease persistence. A recent publication in support of this hypothesis suggests how ubiquitination of one of the proT-enriched proline codon proteins belonging to the PE-PGRS family was identified as a signal for the host to eliminate the pathogen (65). This information could be used to design a better and more efficient vaccine against tuberculosis. In light of our current findings, it will be very intriguing to study the role of PE-PGRS proteins in modulating the host response and their role in the disease progression during *M. tuberculosis* infection. Furthermore, the functional characterization of proT tRNA-encoded proline-rich proteins and their implications in the stage-specific replication and growth rate of *M. tuberculosis* inside the host should be explored.

The findings support our hypothesis that the VapC12 toxin acts as a molecular switch that regulates growth in the presence of cholesterol. Because an actively growing *M. tuberculosis* culture is always heterogeneous and has individual bacteria growing at different rates, the rate of growth is directly proportional to the level of the VapBC12 TA protein accumulated in the cytoplasm. Upon exposure to cholesterol, the fate of each bacterium is dictated by the concentration of the activated toxin present inside the cell. Depending on the intracellular levels of the activated toxin, the bacterium is either eliminated or acquires a metabolically less active state. This results in the enrichment of the slow-growing population in *M. tuberculosis* culture exposed to a cholesterol-rich environment (see Fig. S10) in the supplemental material. Furthermore, the extracellular presence of this toxin ensures the clearance of any rapidly dividing mutant bacteria generated due to the spontaneous incorporation of a genetic lesion. This is the first study to identify a novel mechanism of cholesterol-dependent stochastic enrichment of slow-growing *M. tuberculosis* during mycobacterial infection.

10.1128/mSystems.00855-20.10 FIG S10 Graphical abstract of the study indicating degradation of the anti-toxin VapB12 and subsequent activation of the toxin VapC12 under cholesterol-rich condition. The fast-growing bacteria with higher expression of toxin are eliminated or killed compared to the slow-growing bacteria in the population with less expression of the toxin VapC12. Download FIG S10, TIF file, 0.3 MB. Copyright © 2020 Talwar et al. 2020 Talwar et al. This content is distributed under the terms of the Creative Commons Attribution 4.0 International license.

These findings will help to identify a novel mechanism of the generation of antibiotic persistence and define targets against the persister population. Approaches targeting the persister population will enhance the rate of clearance of the pathogen, resulting in a significant reduction in the duration of treatment. This will help in significantly reducing the risk associated with the current extended regimen extending from 6 months to 2 years. Thus, we have empirically demonstrated that both antibiotic and disease persistence contributes to chronic *M. tuberculosis* infection, and targeting pathways essential for both could potentially shorten the treatment regimen. This finding is significant since a better understanding of the disease persistence and targeting the *M. tuberculosis* persister population as a therapeutic strategy will open new paradigms in tuberculosis treatment.

## MATERIALS AND METHODS

### Bacterial strains and culture.

M. tuberculosis mutants were derived from strain H37Rv by using homologous recombination between the suicide plasmid and bacterial genome. Flanking regions (1,000 bp) of the target gene, Rv1720c (*vapC12*) were cloned in pJM1 suicide vector and electroporated in H37Rv-competent cells using standard M. tuberculosis protocols (Tanya Parish and Neil G. Stroker). The strains were maintained on Middlebrook 7H11 agar or 7H9 broth (Difco Middlebrook 7H11 agar [catalog no. 283810] and 7H9 broth [catalog no. 271310]) supplemented with 10% OADC enrichment. Hygromycin was added at 50 μg/ml. To complement the *vapC12* mutant, the *loxP*-flanked chromosomal hygromycin resistance gene was excised by the expression of Cre recombinase. This strain was transformed with pJEB402 harboring the Rv1720c-1721c (*vapBC12*) genes. For growth on defined carbon sources, strains were grown in “minimal media” (0.5 g/liter asparagine, 1 g/liter KH_2_PO_4_, 2.5 g/liter Na_2_HPO_4_, 50 mg/liter ferric ammonium citrate, 0.5 g/liter MgSO_4_⋅7H_2_O, 0.5 mg/liter CaCl_2_, 0.1 mg/liter ZnSO_4_) containing 0.1% (vol/vol) glycerol or 0.01% (wt/vol) cholesterol and 50 mg/ml sodium palmitate. Growth was determined by CFU plating at different time points on 7H11 with 10% OADC plates. Similar protocols were followed for maintaining and culturing M. bovis BCG strains

### Growth curve.

Log-phase cultures of wild-type H37Rv, *ΔvapC12*, and *ΔvapC12:vapBC12* strains were washed with phosphate-buffered saline plus Tween (PBST) twice and inoculated in minimal media with 0.1% glycerol and 0.01% cholesterol, respectively, at an absorbance of 0.005. Aliquots of the cultures were obtained at different time points and plated on 7H11+OADC plates for bacterial enumeration. Similar protocols were followed for M. bovis BCG strains.

### Resazurin-based metabolic activity assay.

Log-phase cultures of wild-type H37Rv, *ΔvapC12*, and *ΔvapC12:vapBC12* strains at an optical density (OD) of 0.5 were washed with PBST twice, and the OD at 600 nm (OD_600_) was set to 0.05 in glycerol and cholesterol media. These cultures were serially diluted in the respective media in a 96-well plate. The experiment was performed in duplicate, and both plates were incubated at 37°C for 5 days before PrestoBlue cell viability reagent (Invitrogen catalog no. A13261) was added to each well in one set of the plates. The plates were incubated for another 2 days. The fluorescence read-out of the plate with PrestoBlue was taken at 570/585 nm using a Synergy HTX multi-mode microplate reader. For bacterial enumeration in each well, CFU plating was done with a plate with no PrestoBlue. To determine the average metabolic activity, the total fluorescence recorded was normalized for the number of bacteria in the corresponding well.

### Antibiotic kill curve.

Log-phase cultures of wild-type M. bovis BCG, BCG *vapC12* mutant, and BCG *ΔvapC12:vapBC12* strains grown in 7H9-enriched media were washed with PBST twice and inoculated in glycerol and cholesterol media at an absorbance of 0.05. The cultures were allowed to grow for 4 days before being treated with 5× MIC of rifamycin. Bacterial enumeration was performed using CFU plating of cultures on 7H11+OADC plates at various time points. The kill curve was plotted by calculating the percent survival.

### *In vitro* stress assay.

Log-phase cultures of wild-type H37Rv and *vapC12* mutant strains were washed with PBST twice and inoculated at an absorbance of 0.1 in 7H9-enriched media for each stress condition, keeping an untreated control. The survival was plotted by CFU plating at different time points posttreatment for different stress conditions, *viz.*, oxidative (5 mM H_2_O_2_ for 6 h) and nitrosative (200 μM DETA-NO for 24 h).

### Bone marrow-derived macrophages.

Bone marrow-derived macrophages (BMDM) were isolated by culturing bone marrow cells from C57BL6 mice in Dulbecco modified Eagle medium containing 10% fetal bovine serum, 2 mM glutamine, 10% L929-conditioned media, and 10 μg/ml ciprofloxacin for 5 days. Approximately 24 h prior to infection, differentiated BMDM were detached and seeded on a 24-well tissue culture plate at 5 × 10^5^ cells/well in the same medium lacking antibiotic. Macrophages were infected with different strains of M. tuberculosis at an MOI of 1 for 4 h at 37°C and 5% carbon dioxide. Extracellular bacteria were removed by three washes with warm PBS. Intracellular bacteria were quantified by lysing the cells with 0.01% Triton X-100 (Sigma, CAS:9002-93-1) at the indicated time points and plating dilutions on 7H11 agar.

### RNA sequencing.

Log-phase cultures of H37Rv and *ΔvapC12* were washed with PBST twice and inoculated in glycerol and cholesterol media at an absorbance of 0.005. RNA was isolated from the cultures at day 4 using an RNeasy minikit according to the manufacturer’s protocols (Qiagen, catalog no. 74104). The RNA was DNase treated using a Turbo DNA free kit according to the manufacturer’s protocol (Thermo Fisher Scientific) to remove any genomic DNA contamination. All mycobacterial total RNAs were analyzed using an Agilent Bioanalyzer (Agilent, Santa Clara, CA) for quality assessment with an RNA integrity number (RIN) range of 5.6 to 9.7 and a median of 7.5. rRNA was depleted from 500 ng of bacterial RNA using a RiboMinus bacterial transcriptome isolation kit (Invitrogen/Thermo Fisher Scientific, Waltham, MA) according to the manufacturer’s protocol. cDNA libraries were prepared from the resultant rRNA-depleted RNA and 1 μl of a 1:500 dilution of ERCC RNA Spike in Controls (Ambion/Thermo Fisher Scientific) using Lexogen SENSE total RNA-seq Library Prep kit (Lexogen GmnH, Vienna, Austria) according to the manufacturer’s protocol, except with 21 PCR cycles. The length distribution of the cDNA libraries was monitored using a DNA high-sensitivity reagent kit on a Perkin-Elmer Labchip (Perkin-Elmer, Waltham, MA). All samples were subjected to an indexed paired-end sequencing run of 2 × 51 cycles on an Illumina HiSeq 2000 system (Illumina, San Diego, CA; 16 samples/lane). Raw reads (FASTQ files) were mapped to the M. tuberculosis H37Rv (GenBank accession number AL123456) using bowtie2 with the default parameters. The gene counts were then counted using featureCounts (part of the Subread package) using the genome annotations provided in the GenBank file. The gene counts were then used in DESeq2 for differential gene expression analysis. Multiple testing correction was performed using the method of Benjamini and Hochberg. *P* values of <0.05 were deemed statistically significant. Computations were done using the R statistical language, v3.3.1.

### Quantitative RT-PCR.

Comparative qRT-PCR was performed on RNA isolated from various strains under different experimental conditions. RNA isolation was done from culture using an RNeasy minikit according to the manufacturer’s protocols (Qiagen, 74104). The RNA was DNase treated using a Turbo DNA-free kit according to the manufacturer’s protocol (Thermo Fisher Scientific) for making cDNA with an Accuscript Hi-Fidelity cDNA synthesis kit (Agilent). The qRT-PCR was set up using Brilliant III Ultra-Fast SYBRgreen qPCR master mix in an Mx3005P qPCR system (Agilent). The primers used are listed in Table 4. The data analysis was done using MxPro software.

**TABLE 4:** Primers used in this study

Primer	Sequence (5′–3′)
Rv1720-F1	ATGATATCTGGACTTGTCGATCCTGGAC
Rv1720-R1	ATGCGGCCGCAATGCGGAGATCGAGCTTGTC
Rv1720-F2	ATGGGCCCCGAGGCGTCCAACACGAT
Rv1720-R2	ATGGGCCCGCAATCCGCGCACAAAGAAC
1720-conf1	GGAGATGCACCCGTTCTTGAC
1720-conf2	ATGACCTTGATTTCCGGCTGCC
1720-21-F	GCTTTCGAATTAATTAAATGTCCGCCATGGTTCAGATCC
1720-21-R	CAGATTTAAATTCAGGCGACAAGCTCGATCTC
1721-F-P	CTCGTTAATTAAATGTCCGCCATGGTTCAGATCC
1721-R-S	CAGATTTAAATTCACTCAGATCGAGCCTCGTC
T7proU	TCGGGGTGACAGGATTTGAACCTGCGGCCTTCCGCTCCCAAAGCGGATGCGCTACCAAGCTGCGCTACACCCCGCCTATAGTGAGTCGTATTA
T7proT	TCGGGCTGACAGGATTTGAACCTGCGACCACTTGACCCCCAGTCAAGTGCGCTACCAAACTGCGCCACAGCCCGCCTATAGTGAGTCGTATTA
T7 top strand	TAATACGACTCACTATAGG
T7proT cg-2	TCGGGCTGACAGGATTTGAACCTGCGACCACTTGAGCCCCAGTCAAGTGCGCTACCAAACTGCGCCACAGCCCGCCTATAGTGAGTCGTATTA
T7proT cg-3	TCGGGCTGACAGGATTTGAACCTGCGACCACTTGACGCCCAGTCAAGTGCGCTACCAAACTGCGCCACAGCCCGCCTATAGTGAGTCGTATTA
T7proT gc-5	TCGGGCTGACAGGATTTGAACCTGCGACCACTTGACCCCCACTCAAGTGCGCTACCAAACTGCGCCACAGCCCGCCTATAGTGAGTCGTATTA
T7proT at-4	TCGGGCTGACAGGATTTGAACCTGCGACCACTTGACCCCCTGTCAAGTGCGCTACCAAACTGCGCCACAGCCCGCCTATAGTGAGTCGTATTA
T7proT pU-6	TCGGGCTGACAGGATTTGAACCTGCGACCACTTGACCCCAAGTCAAGTGCGCTACCAAACTGCGCCACAGCCCGCCTATAGTGAGTCGTATTA
RpfA F	AATTCGAAATGCATCATCACCACCACCATATGAGTGGACGCCACCGTAAG
RpfA R	AAGTTAACTCAGCCGATGACGTACGGCT
Rv1468 F	AATTCGAAATGCATCATCACCACCACCATATGTCGTTCGTGGTCGCGAATAC
Rv1468 R	AAGTTAACCTATGTTCCGTTCGCGCCG
Rv2340 F	AATTCGAAATGCATCATCACCACCACCATATGTCGCACGTTACCGCGG
Rv2340 R	AAGTTAACTCATTCGTGCCCGGGCG
Rv3097 F	AATTCGAAATGCATCATCACCACCACCATATGGTGTCTTATGTTGTTGCGTTGC
Rv3097 R	AAGTTAACTCAGGCGGCGATACCGAGTT
Rv1068 F	AATTCGAAATGCATCATCACCACCACCATATGTCCTACATGATTGCGGTGCC
Rv1068 R	AAGTTAACTTATTGCCCGGGCGTGCC
Rv1441 F	AATTCGAAATGCATCATCACCACCACCATATGTCGAACGTGATGGTAGTCCC
Rv1441 R	AAGTTAACTCACCCGTGCTTTCCTTGCG
1720-21His F	GCCTTCGAAATGCATCATCACCACCACCATATGTCCGCCATGGTTCAGATCCGCAACGTTCCCG
1720-21 R	CAGATTTAAATTCAGGCGACAAGCTCGATCTC
1720-21 F	GCCTTCGAAATGTCCGCCATGGTTCAGATCCGCAACGTTCCCG
1720-21 Flag R	ATTGTTAACTTACTGTCGTCGTCGTCCTTGTAGTCGATGTCGTGGTCCTTGTAGTCACCGTCGTGGTCCTTGTAGTCGGCGACAAGCTCGATCTCCGCATTATGGCCATGGG
b-Actin:S RT	S: CCA ACT GGG ACG ACA TGG AG
b-Actin:A RT	A: CGTAGCCCTCGTAGATGGGC
GAPDH-F RT	ACCACAGTCCATGCCATCAC
GAPDH-R RT	TCCACCACCCTGTTGCTGTA
IFNy-F RT	GACCTGAGCAAGACCCTGAG
IFNy-R RT	GCCATTTCGCCTGACATATT
TNFa-F RT	ATCTACCTGGGAGGCGTCTT
TNFa-R RT	GAGTGGCACAAGGAACTGGT
IL-1b-F RT	GGGCCTCAAGGGGAATC
IL-1b-R RT	GAGCACCCCTTAGCGTGCTCT
IL-10-F RT	GGCACGAACACCCAGTCTGA
IL-10-R RT	TCACCTGCTCCACTGCCTTG
IL-12p40-F RT	TCTGAGCCGGTCACAACTGC
IL-12p40-R RT	AGGCGCTGTCCTCCTGACAC
Inos-F RT	GCACACGTTGGCTTCCCTCT
Inos-R RT	TGGGCCAGTGCTTCTGATTTTCC
GM-CSF-F RT	CTGTGGTTTGCAGCATCTGT
GM-CSF-R RT	GGGGCTCAAACTGGTCATAG
IL-2-F RT	CTTCAAGCTCTCCAAAGCA
IL-2-R RT	CCATCTCTTCAGAAATTCCAC
TGF-β-F RT	CGGGGCCTGGACACCAACTATTGC
TGF-β-R RT	CTGCTCCACCTTGGCTTTGCGGCCCAC
proU RT F	CGGGGTGTAGCGCAGCTT
proU RT R	TCGGGGTGACAGGATTTGAACCT
proT RT F	CGGGCTGTGGCGCAGT
proT RT R	TCGGGCTGACAGGATTTGAACC
proY RT F	CGGGGTGTGGCGCAG
proY RT R	TCGGGGTGGCGGGATTTG
SigH RT F	TACTGACCAACACCTACATCA
SigH RT R	CGGCAACGCTTCTAACGCTTC
arg T RT F	GCCCTCGTAGCTCAGGG
arg T RT R	TGCCCCCGGCAGGATTC
argV RT F	GCCCCCGTAGCTCAGG
argV RT R	TGCCCCCGGCAGGATTC
argU RT F	GCGCCCGTAGCTCAACG
argU RT R	CGCGCCCGAAGAGATTCGAA
glyU RT F	GCCGATGTAGTTCAATGGCAGAAC
glyU RT R	AGCCGATGACGGGAATCGAAC
glyV RT F	GCGGGCGTAGCTCAATGGT
glyV RT R	AGCGGGCGACGGGAATC
glyT RT F	GCGGATGTAGCGCAGTTGGT
glyT RT R	AGCGGATGACGGGATTCGAAC
alaV RT F	GGGGCTATGGCGCAGTTG
alaV RT R	TGGAGCTAAGGGGATTCGAACC
alaU RT F	GGGGCTATGGCGCAGCT
ala U RT R	TGGAGCTAAGGGGACTCGAAC
1721-K-A-F	GCTGGCGGCCCGC
1721-K-A-R	GGGCCGCCAGCTCGT
Rv1720 d5 F	GTGATCGTGTTGGCCGCC
Rv1720 d5 R	CGCCGAGGCGGCCA
Rv1720 d94 F	GCCGCTGGAGCCTACG
Rv1720 d94 R	GACGTAGGCTCCAGCGG

### VapC12 expression and protein purification.

*vapC12* (Rv1720c) was cloned in pET28a vector using the primer sequences listed in Table 4. The SDM mutants D_5_A and D_94_A were generated by DpnI treatment. The clones were transformed in E. coli Rosetta cells. Briefly, the overnight culture was inoculated into 1 liter of fresh LB media (1:100) supplemented with 100 μg/ml of kanamycin and allowed to grow until the OD_600_ reached ∼0.5. The culture was then induced with 1 mM IPTG (isopropyl-β-d-thiogalactopyranoside) and allowed to grow overnight at 37°C. Cells were harvested by centrifugation at 6,000 × *g* for 10 min and checked for expression of wild-type or mutant rRv1720c by SDS-PAGE. Most of the target protein was present in the pellet as inclusion bodies (IBs). Isolation of pure IBs containing rRv1720c was performed by sonication and several washing steps. Briefly, purified rRv1720c IBs (1 ml) were solubilized in 9 ml of buffer (50 mM Tris-HCl [pH 8.0], 300 mM NaCl, 10 mM β-mercaptoethanol, 8 M urea) and incubated at room temperature for 1 h, followed by centrifugation at 15,000 × *g* for 20 min at 10°C. The supernatant obtained after centrifugation was used for the purification of recombinant Rv1720c protein by immobilized metal ion affinity chromatography using a HisTrap FF column (GE Healthcare Buckinghamshire, UK) under denaturing conditions. Protein was eluted using buffer (50 mM Tris-HCl [pH 8.0], 300 mM NaCl, 10 mM β-mercaptoethanol, 8 M urea, 250 mM imidazole). Denatured purified protein was refolded by dilution in a pulsatile manner in refolding buffer (100 mM phosphate buffer [pH 6.4], 300 mM NaCl, 5 mM β-mercaptoethanol) at 4°C with constant stirring. The refolded target protein sample was centrifuged at 24,000 × *g* for 30 min at 4°C, and the supernatant containing refolded active protein was concentrated and dialyzed three times against buffer (100 mM phosphate buffer [pH 6.4], 300 mM NaCl, 5 mM β-mercaptoethanol). The final buffer exchange of protein to buffer (100 mM phosphate buffer [pH 6.4], 300 mM NaCl, 10% glycerol) was performed by PD10 desalting column (GE Healthcare Buckinghamshire, UK) according to the manufacturer’s protocol. Protein was quantitated by a bicinchoninic acid assay (Thermo Scientific Pierce, Rockford, IL), analyzed by SDS-PAGE, and confirmed by Western blotting with anti-His monoclonal antibody (Cell Signaling Technology, Inc., Danvers, MA). A similar protocol was followed for purification of rRv1720cD_5_A and rRv1720cD_94_A.

Also, *vapB12* (Rv1721c) cloned in pET28a E. coli *Rosetta* cells was purified from the supernatant of lysed induced cells using a HisPur Cobalt purification kit (3 ml; Thermo Scientific, catalog no. 90092).

### *In vitro* transcription of tRNA.

The tRNAs were transcribed using a MegaScript kit (Invitrogen, AM1334) according to the manufacturer’s protocol, which was followed by phenol-chloroform extraction and isopropanol precipitation for purified transcript. The primers used for *in vitro* transcription are listed in Table 4.

### *In vitro* tRNA cleavage assay.

*In vitro* RNA cleavage assay was performed using tRNAs produced via T7 transcription. Cleavage reactions using 3 pmol of tRNA were incubated with 30 pmol of recombinant proteins (rRv1720c, rRv1720cD_5_A, and rRv1720cD_94_A) and at 37°C for 3 h in tRNA cleavage buffer (10 mM HEPES [pH 7.5], 15 mM potassium chloride, 3 mM magnesium chloride, 10% glycerol). The samples were mixed with 2× formamide gel loading buffer (95% [wt/vol] formamide, 50 mM EDTA) and incubated at 95°C for 5 min before running on a 3% agarose gel. The bands were visualized by using ethidium bromide and exposing the gel to UV light.

### Persister enrichment assay.

Log-phase cultures of wild-type H37Rv and *ΔvapC12* strains (Table 5) were washed with PBST, and an OD of 0.005 was set in 7H9-enriched media, glycerol media, and cholesterol media. The cultures were washed after 4 days, and fresh cholesterol medium was added to all the tubes. Bacterial enumeration was done by CFU plating on 7H11+OADC plates at different time points. A similar protocol was followed for M. bovis BCG strains.

**TABLE 5:** Strains used in this study

Strain or vector	Host	Marker	Tag	Source
H37Rv				Christopher M. Sassetti
BCG (*M. bovis* Danish)				ATCC
E. coli XL-1 Blue		Tetracycline		Stratagene
pET28a		Kanamycin		Novagen
pUV15tetO		Hygromycin		Sabine Ehrt
pMV261		Kanamycin		Christopher M. Sassetti
E. coli Rosetta				Novagen
Rv1720pET28a	Rosetta DE3	Kanamycin		
Rv1720 D5:A pET28a	Rosetta DE3	Kanamycin		
Rv1720 D94:A pET28a	Rosetta DE3	Kanamycin		
ΔRv1720c	H37Rv	Hygromycin		
ΔRv1720c:1720-21pJEB402	H37Rv	Hygromycin-kanamycin		
BCGΔ1720c	BCG	Kanamycin		
BCG: 1468c pMV261	BCG	Kanamycin	His	
BCG: 1068c pMV261	BCG	Kanamycin	His	
BCG: 1441c pMV261	BCG	Kanamycin	His	
BCG: 2340c pMV261	BCG	Kanamycin	His	
BCG: 3097c pMV261	BCG	Kanamycin	His	
BCGΔ1720c:1468c pMV261	BCG	Hygromycin-kanamycin	His	
BCGΔ1720c:1068c pMV261	BCG	Hygromycin-kanamycin	His	
BCGΔ1720c:1441c pMV261	BCG	Hygromycin-kanamycin	His	
BCGΔ1720c:2340c pMV261	BCG	Hygromycin-kanamycin	His	
BCGΔ1720c:3097c pMV261	BCG	Hygromycin-kanamycin	His	
BCG:Rv1721His-1720 pMV261	BCG	Hygromycin-kanamycin	His	
BCG:Rv1721-1720 flag pMV261	BCG	Hygromycin-kanamycin	3×Flag	
ΔRv0665	H37Rv	Hygromycin		
BCG:1720 pUV15 tetO	BCG	Hygromycin		
BCG:1721 pUV15 tetO	BCG	Hygromycin		
BCG:1720-21 pUV15 tetO	BCG	Hygromycin		
BCG:1721His K:A-1720	BCG	Kanamycin	His	
BCGΔ1720:1721 His K:A-1720	BCG	Kanamycin	His	

### Spent medium preparation.

Log-phase cultures of H37Rv and *ΔvapC12* were washed with PBST and inoculated at an OD of 0.005 in cholesterol media. The cultures were then grown for a week at 37°C in an incubator shaker. The cultures were pelleted down, and the supernatant was filtered through a 0.2-μm filter. The spent cholesterol supernatants of both strains were used for further experiments. To prepare heat-inactivated supernatant, the spent medium was exposed to 95°C for 30 min before being added to the cultures. A similar protocol was followed for carrying out the experiment in BCG strains.

### Spent medium experiment.

Log-phase cultures of *ΔvapC12* were washed and inoculated in cholesterol media at an OD of 0.005 in triplicates. The cultures were grown for 4 days and then washed with PBST and resuspended in spent H37Rv cholesterol supernatant, H37Rv cholesterol spent supernatant media supplemented with purified toxin or antitoxin, or *ΔvapC12* cholesterol spent supernatant according to the experiment, and fresh cholesterol. Bacterial enumeration was done by CFU plating on 7H11+OADC plates at different time points. A similar protocol was followed for the experiment in the M. bovis BCG strain.

### Culture filtrate experiment.

*vapB12* (antitoxin) and *vapC12* (toxin) were cloned as an operon in pMV261 kanamycin vector with a C-terminal flag tag using primers listed in Table 4. The clone was electroporated in M. bovis BCG and maintained in 7H9-enriched media. A log-phase culture of C-terminal flag-tagged BCG:*vapBC12* was washed with PBST twice, inoculated into glycerol and cholesterol media at an OD of 0.5, and allowed to grow for 48 h. Cultures were pelleted down, and the supernatant was filtered through a 0.2-μm filter. The supernatant was precipitated with 5% trichloroacetic acid overnight at 4°C and centrifuged in Oak Ridge tubes at 12,000 rpm for 20 min at 4°C. The pellet thus obtained was washed twice with ice-cold acetone, allowed to dry, and resuspended in 1× Laemmli buffer. The samples were run on 15% PAGE and developed with the rabbit anti-flag antibody (Sigma, F7425). The samples were also blotted against the Ag85B antibody (Abcam, ab43019) and Hsp65 antibody (kindly provided by Vinay K. Nandicoori) as positive and negative controls, respectively.

### Antitoxin degradation experiment.

The *vapBC12* operon with an N-terminal His tag was cloned in pMV261 vector. The clone was electroporated in M. bovis BCG. The culture was maintained in 7H9-enriched media. A log-phase culture was washed with PBST twice and inoculated in glycerol and cholesterol media at an OD of 0.5. Aliquots of culture were taken out, washed, and lysed in PBS at different time points (4, 6, 24, and 48 h) postinoculation. The lysates were run on a 15% PAGE gel and developed using monoclonal anti-His antibody (Biospecs, BTL1010) with Super Signal West Femto maximum-sensitivity substrate (Thermo Scientific, catalog no. 34095).

### PE-PGRS/RpfA blots.

All selected PE-PGRS genes, along with the *rpfA* (with high proT and high proY codon usage), were cloned in pMV261 using primers listed in Table 4. The proteins were translationally fused with His tag at the N-terminal by incorporating bases encoding His tag in the forward cloning primers. The clones were transformed in BCG, and the cultures were maintained in 7H9-enriched media. The log-phase cultures of the constructs were washed twice with PBST and inoculated at an OD of 0.005 in glycerol and cholesterol media. The cultures were allowed to grow until reaching an OD of 0.8 and then pelleted, washed, and lysed in PBS. The samples were run on a 10% SDS-PAGE gel and developed with anti-His antibody (Biospecs, BTL1010).

### *In vivo* animal experiments.

The animal experiments were approved by the animal ethics committee of ICGEB (approval IAEC/THSTI/2015-1). The animal experiments were performed in accordance with the guidelines of the Committee for Purpose of Control and Supervision of Experiments on Animals (CPCSEA, Government of India). Three- to four-week-old female Dunkin-Hartley guinea pigs (200 to 300 g) were used to check the pathogenicities of various *M. tuberculosis* strains. The guinea pigs were infected with various *M. tuberculosis* strains via the aerosol route with 100 bacilli isolated from the log-phase culture (OD = 0.8 to 1). The bacterial load was estimated by CFU plating at different time points postinfection. Briefly, animals were sacrificed at day 1, week 4, and week 7 postinfection. Infected lungs and spleens were harvested and homogenized using a tissue homogenizer. After serial dilution, these tissue lysates were plated on 7H11+OADC plates. For histopathological analysis, lung sections were fixed in 10% formalin and stained with hematoxylin and eosin. The tissue samples were coded and evaluated for the granulomatous organization by a pathologist who had no prior knowledge of the samples. All granulomas in each section were scored, and the scores were added to obtain a total granuloma score. In addition, sections were semiquantitatively assessed for the percentage of the section occupied by granuloma, which was expressed as the granuloma fraction.

### Cytokine profiling and RNA isolation from the spleen.

Single-cell suspension of splenocytes was prepared by passing the spleen through a cell strainer (0.45 μm). The back of the syringe plunger was used to macerate the cells through the filter. The cell pellet was incubated with the RBC lysis buffer for 4 to 5 min after, and then the pellet was used to extract RNA. RNA was isolated from both uninfected and infected spleens harvested from guinea pigs at 7 weeks postinfection using an RNeasy minikit according to the manufacturer’s protocol. cDNA synthesis and qRT-PCR were performed as described previously using primers listed in Table 4.

### Lysine SDM generation.

The SDM *vapB12* mutant, wherein K_19_ (lysine) was mutated to A_19_ (alanine), was generated by DpnI enzyme treatment. The *vapBC12*His pMV261kan plasmid was used as a template for PCR amplification using SDM primers listed in Table 4. The PCR product was PCR purified and treated with DpnI enzyme for 4 h at 37°C. No-template and no-DpnI-treatment control samples were also obtained. All the reactions were transformed in *E. coli* XL-1 Blue-competent cells. The construct was sequenced and electroporated in M. bovis BCG-competent cells.

### Immunoprecipitation.

BCG:*vapBC12* His-tagged pMV261 and BCG:*vapB_K19A_C12* His-tagged cultures were maintained and grown in 7H9-enriched media, and log-phase cultures of the strains were washed twice with PBST and resuspended in glycerol and cholesterol media at an OD of 0.1. After 48 h, the cultures were pelleted and lysed in 1× PBS. The protein quantification of the lysates was done using a Pierce BCA protein assay kit (Thermo Fisher Scientific) according to the manufacturer’s protocol. A 1-mg portion of the lysate was incubated with a 1:200 dilution of mouse anti-His antibody (Biospecs, BTL1010), followed by incubation overnight at 4°C on a rocker. The Ag-Ab complex was incubated with protein G-agarose beads and kept at 4°C on a rocker for 8 to 10 h. After the incubation, the supernatant was collected and beads were washed three times with 1× TBST. The final elution was done with 100 mM glycine (pH 2.0), which was neutralized later with Tris-Cl (pH 8.8). The eluted samples were run on the gel and blotted with rabbit anti-His (Santa Cruz, H-15:sc-803) and rabbit anti-acetylated lysine antibody (Cell Signaling, 9441L).

### Sample processing protocol for mass spectrometry.

BCG strain overexpressing the toxin-antitoxin (*vapBC12*) locus where antitoxin is His tagged was used for sample preparation. A log-phase culture of the overexpressed strain was washed with PBS and inoculated into minimal medium with 0.1% glycerol and minimal medium with 0.01% cholesterol. The cultures were allowed to grow for 48 h, and cell lysate was prepared. Immunoprecipitation was performed with an anti-His antibody (BTL1010).

In-solution digestion was carried out using 10 μg of the proteins from each condition. The samples were subjected to reduction and alkylation using 5 mM dithiothreitol (60°C for 45 min) and alkylation using 10 mM iodoacetamide. Trypsin (Gold Mass-Spectrometry Trypsin; Promega, Madison, WI) digestion was carried out at 37°C for 10 to 12 h. The peptides were vacuum-dried and stored at −80°C until LC-MS/MS analysis.

### LC-MS/MS analysis.

All fractions were evaluated by using a 5600 Triple-TOF mass spectrometer that was directly linked to a reversed-phase high-pressure liquid chromatography Ekspert-nanoLC 415 system (Eksigent, Dublin, CA). Formic acid (0.1%) in water was used as mobile phase A, and mobile phase B was 0.1% formic acid in acetonitrile. All fractions were eluted from the analytical column at a flow rate of 250 nl/min using an initial gradient elution of 10% B from 0 to 5 min, transitioned to 40% over 120 min, ramping up to 90% B for 5 min, holding 90% B for 10 min, followed by reequilibration of 5% B at 10 min with a total run time of 150 min. Peptides were injected into the mass spectrometer using a 10-μm SilicaTip electrospray PicoTip emitter. Mass spectra (MS) and tandem mass spectra (MS/MS) were recorded in positive-ion and high-sensitivity mode with a resolution of ∼35,000 full-width half-maximum. Before testing samples on the mass spectrometer, calibration of spectra occurred after the acquisition of every sample using dynamic LC-MS and MS/MS acquisitions of 100 fmol of β-galactosidase. The ion accumulation time was set to 250 ms (MS) or 70 ms (MS/MS). The collected raw files spectra were stored in .wiff format.

### MS data analysis.

All raw MS files were searched in Protein Pilot software (v5.0.1; Sciex) with the Paragon algorithm. For Paragon searches, the following settings were used: sample type, identification; cysteine alkylation, iodoacetamide; digestion, trypsin; instrument, 5600 Triple-TOF; species, H37Rv; maximum allowed missed cleavages, 1; search effort, Thorough ID; and result quality, 0.05. Only peptides with a confidence score of >0.05 were considered for further analysis, and bias correction was automatically applied. False discovery rate (FDR) analysis was also performed through a decoy database. Carbamidomethylation (C) was used as a fixed modification. The peptide and product ion tolerance of 0.05 Da was used for searches. The output of this search is a .group file, and this file contains the following information that is required for targeted data extraction: protein name and accession, cleaved peptide sequence, modified peptide sequence, relative intensity, precursor charge, unused Protscore, confidence, and decoy result.

### Codon usage.

The bioinformatic analysis was done for determining the codon usage of proT and proY in each gene belonging to all 10 functional categories. All of the data and codes used for the analysis are available at https://github.com/ddlab-igib/mtb-codon-usage.

### ATP estimation.

Log-phase cultures of M. bovis BCG wild-type and *ΔBCGvapC12* strains were washed with PBST twice and inoculated in 0.1% glycerol and 0.01% cholesterol media at an absorbance of 0.005. Aliquots of the cultures were taken at day 5 for ATP estimation. Then, 1 ml of each of the culture was pelleted down and resuspended in 0.5 ml of PBS, followed by heat lysis of cultures at 98°C for 10 min. ATP was estimated from bacterial lysates using a BacTiter-Glo assay kit from Promega according to the manufacturer’s protocol. The protein estimation was done in the lysate using Pierce BCA protein assay kit according to the manufacturer’s protocol.

### Lipid isolation and analysis.

Wild-type H37Rv, *ΔvapC12*, and *ΔvapC12:vapBC12* strains were grown in 10 ml of 7H9-enriched media. The cells were harvested and resuspended in chloroform-methanol (2:1, vol/vol) and left overnight. This was followed by sequential extraction with chloroform-methanol (1:1, vol/vol) and chloroform-methanol (1:2, vol/vol). All fractions were pooled, dried, and finally resuspended in 200 μl of chloroform-methanol (2:1). Next, 10 μl of each lipid was spotted onto a TLC plate (Silica gel; Sigma). To detect PDIM, plates were developed in petroleum ether-diethyl ether (90:10) and later exposed to iodine vapors.

### Statistical analysis.

Statistical analysis and graph generation were done using Prism 5 software (v5.01; GraphPad Software, Inc., La Jolla, CA). For normally distributed data, an unpaired Student *t* test was performed on the means of at least three independent experiments. For animal experiment data analysis, a Mann-Whitney test was performed. *P* values of <0.05, <0.01, and <0.005 (indicated as *, **, and ***, respectively, in the figures) were considered significantly different.

### Data availability.

The acquired RNA sequencing reads are available for download from the NCBI Gene Expression Omnibus under accession number GSE135952. The MS data obtained here have been submitted to public data repositories. The raw proteomics data have been deposited in the ProteomeXchange Consortium via the PRIDE partner repository under data set identifier PXD022028 (http://www.ebi.ac.uk/pride/archive/projects/PXD022028). The data were also submitted to the Massive database (http://massive.ucsd.edu/ProteoSAFe/status.jsp?task=c9b55cb05765407f9a61c7661cb27cb1) under ID MSV000086278. The bioinformatic analysis and codes used are available on GitHub (https://github.com/ddlab-igib/mtb-codon-usage).
