# Supplementary figures and images for: Role of VapBC12 Toxin-Antitoxin Locus in Cholesterol-Induced Mycobacterial Persistence
Source: mSystems. 2020 Dec 15;5(6):e00855-20. doi: 10.1128/mSystems.00855-20 (PMC7771538; doi:10.1128/mSystems.00855-20)

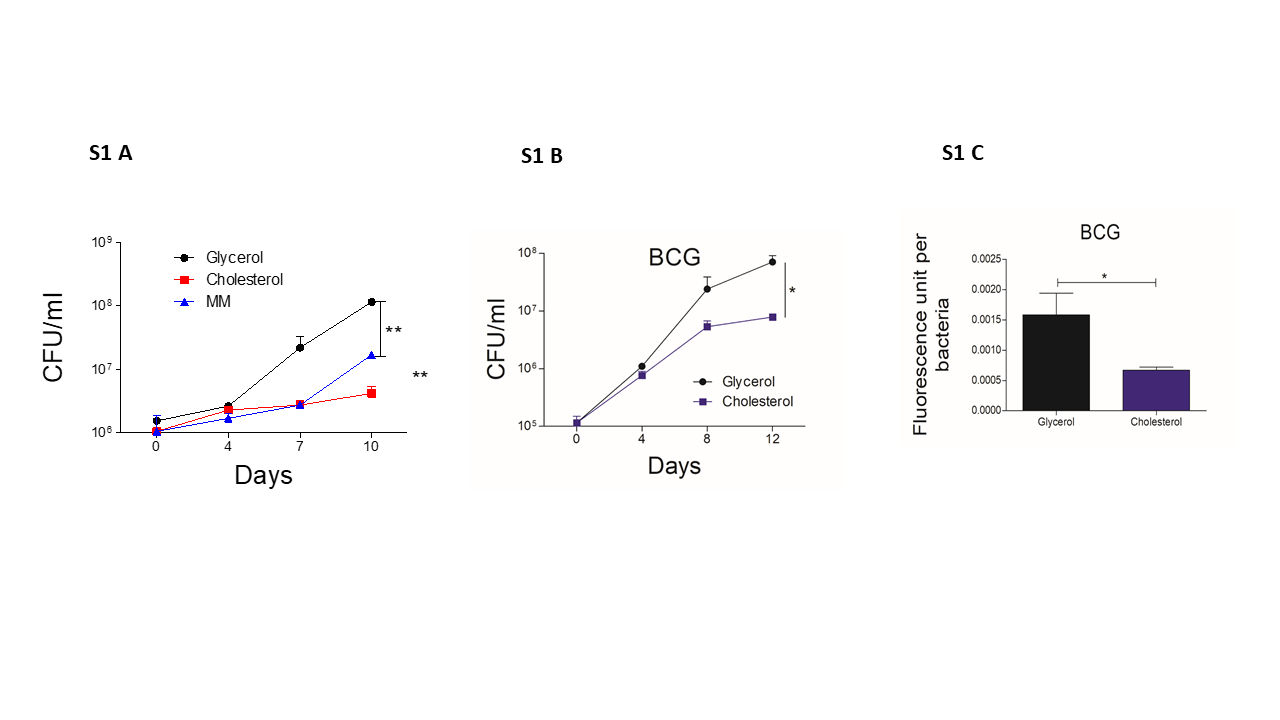

Supplement: FIG S1 [file mSystems.00855-20-sf001.tif]

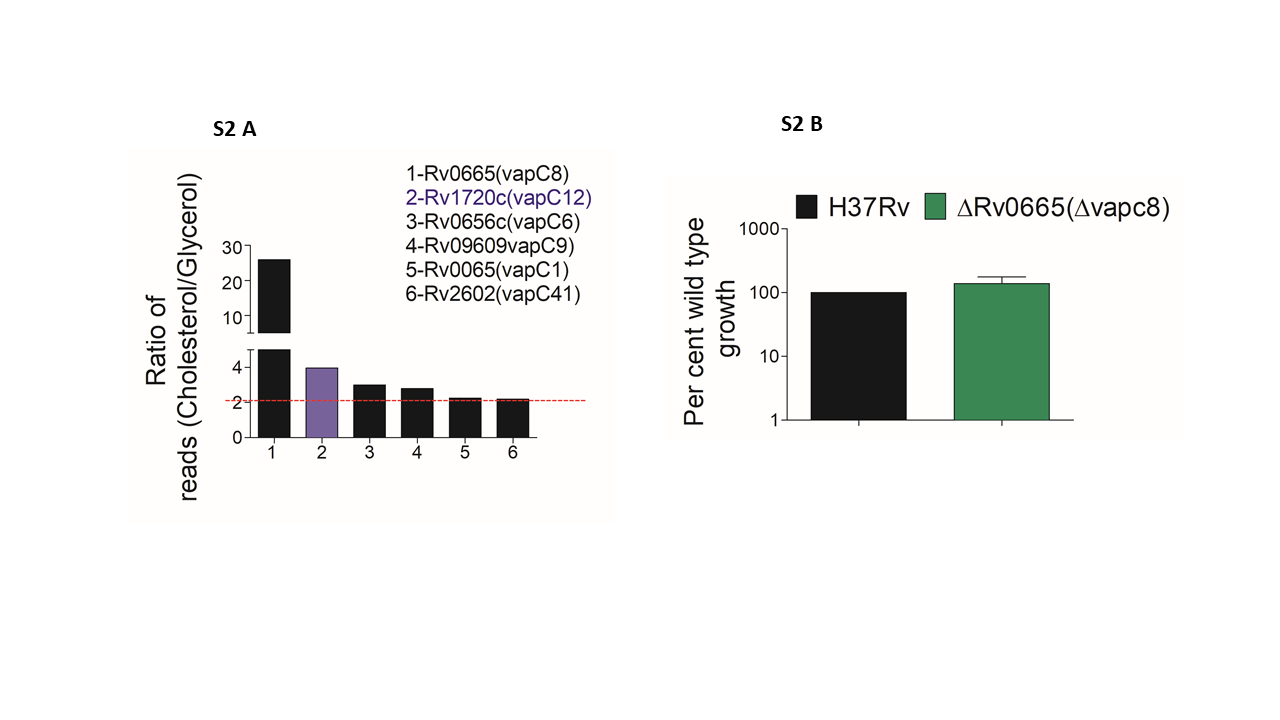

Supplement: FIG S2 [file mSystems.00855-20-sf002.tif]

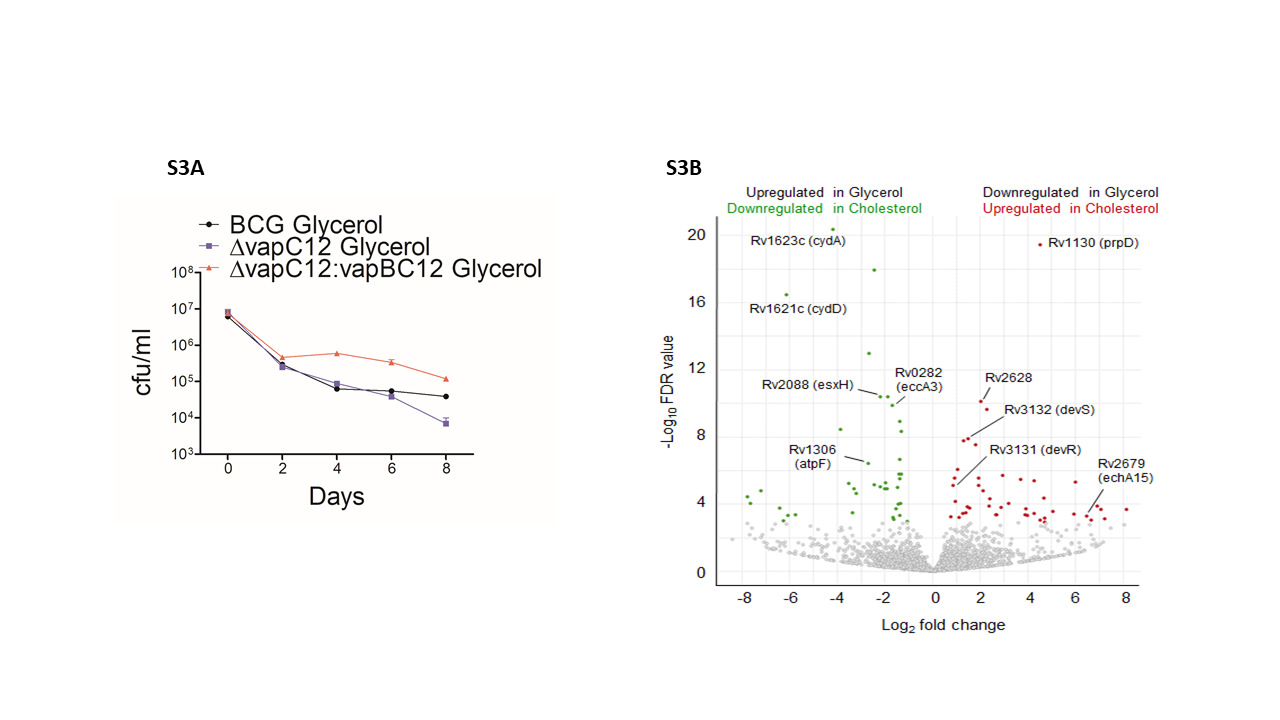

Supplement: FIG S3 [file mSystems.00855-20-sf003.tif]

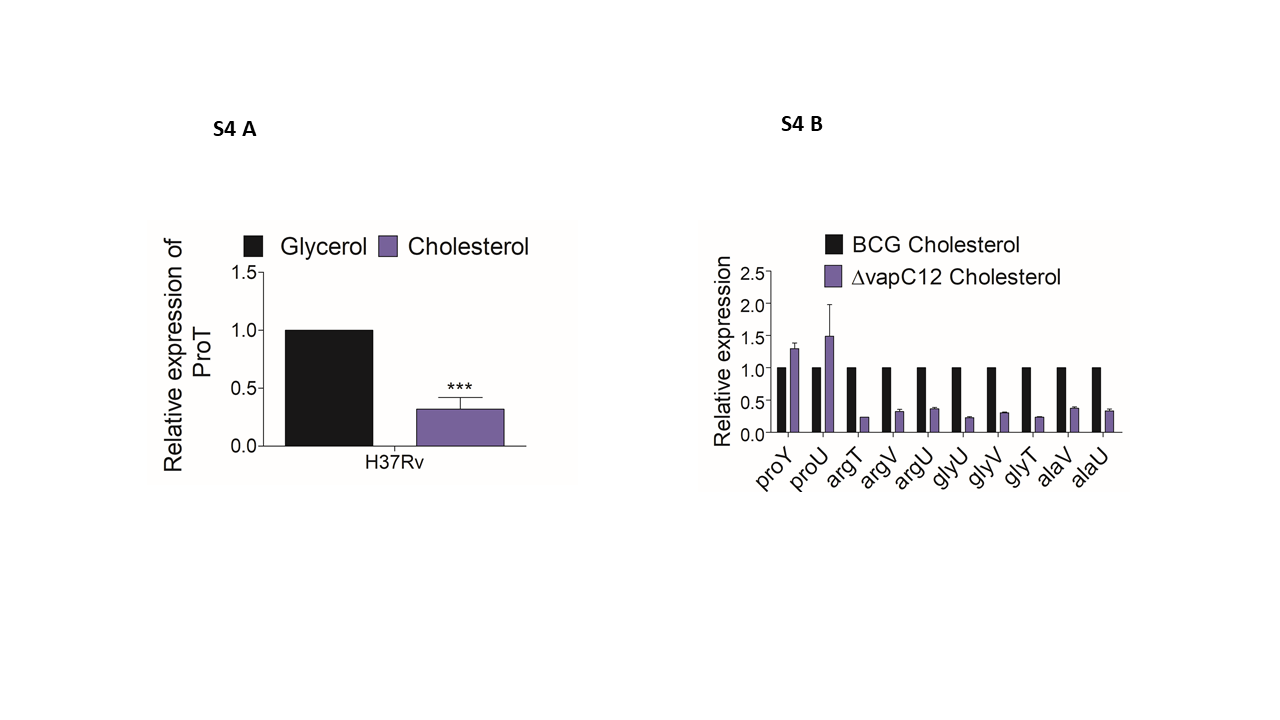

Supplement: FIG S4 [file mSystems.00855-20-sf004.tif]

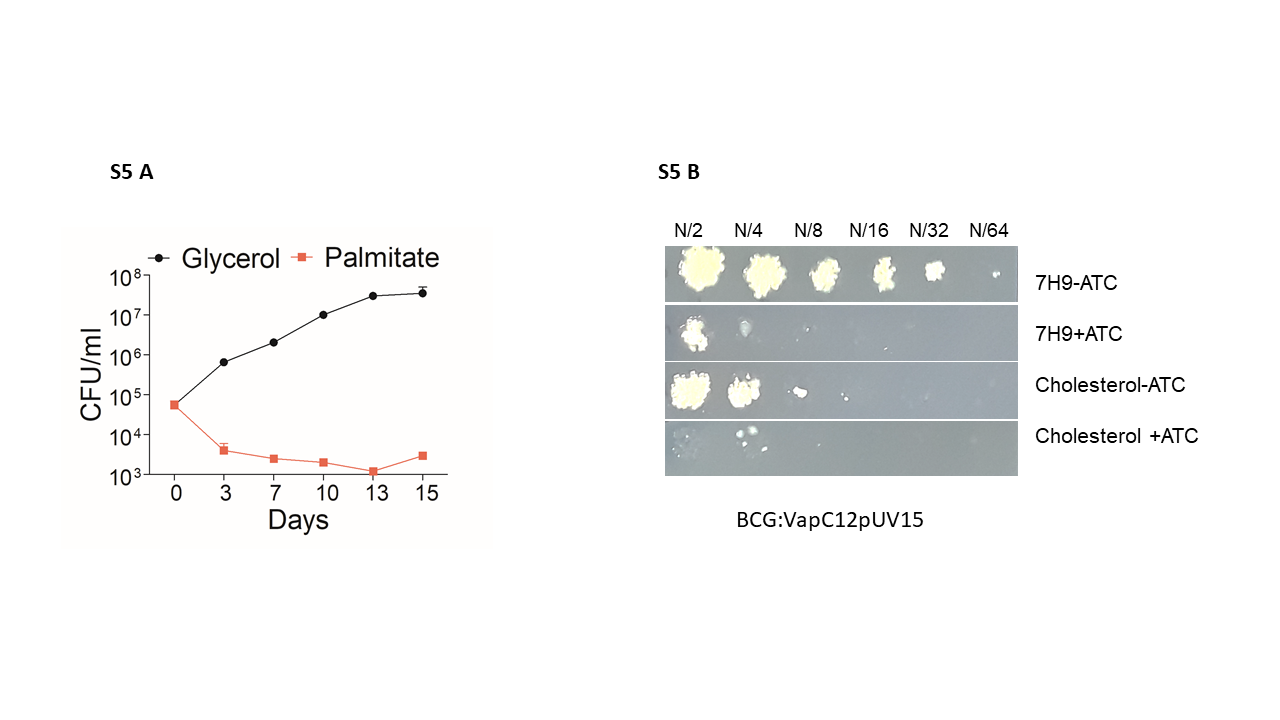

Supplement: FIG S5 [file mSystems.00855-20-sf005.tif]

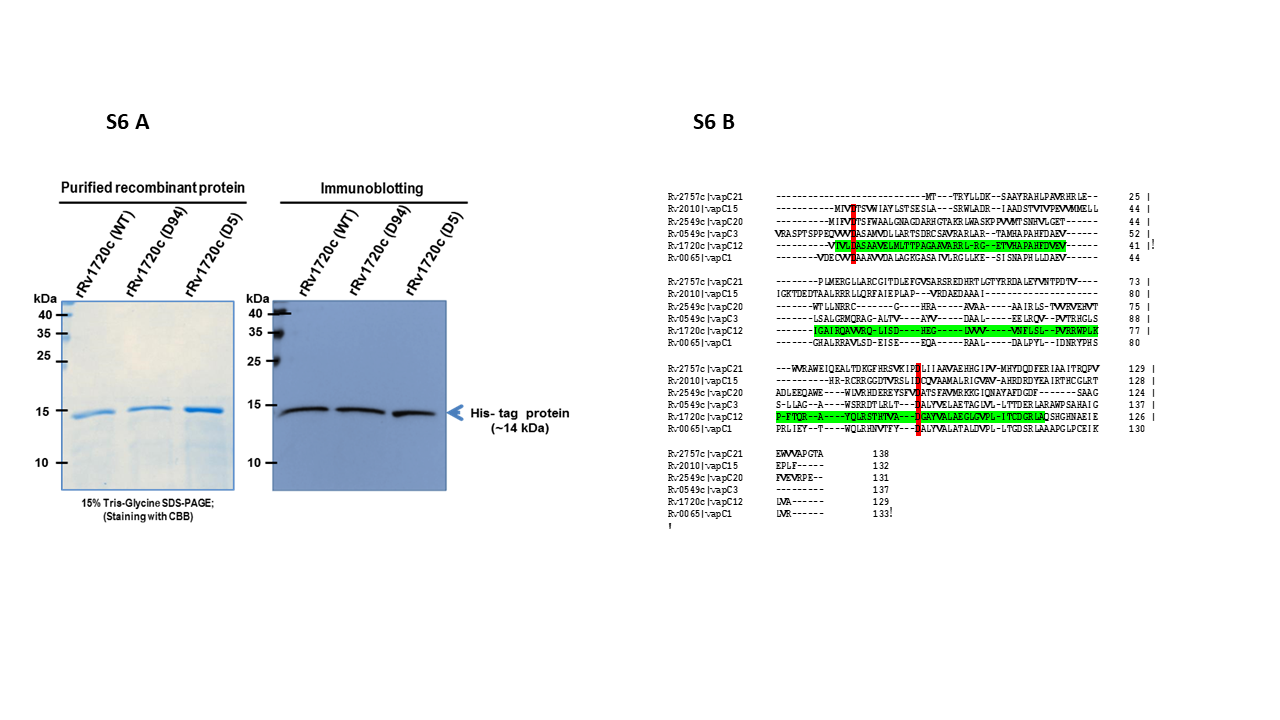

Supplement: FIG S6 [file mSystems.00855-20-sf006.tif]

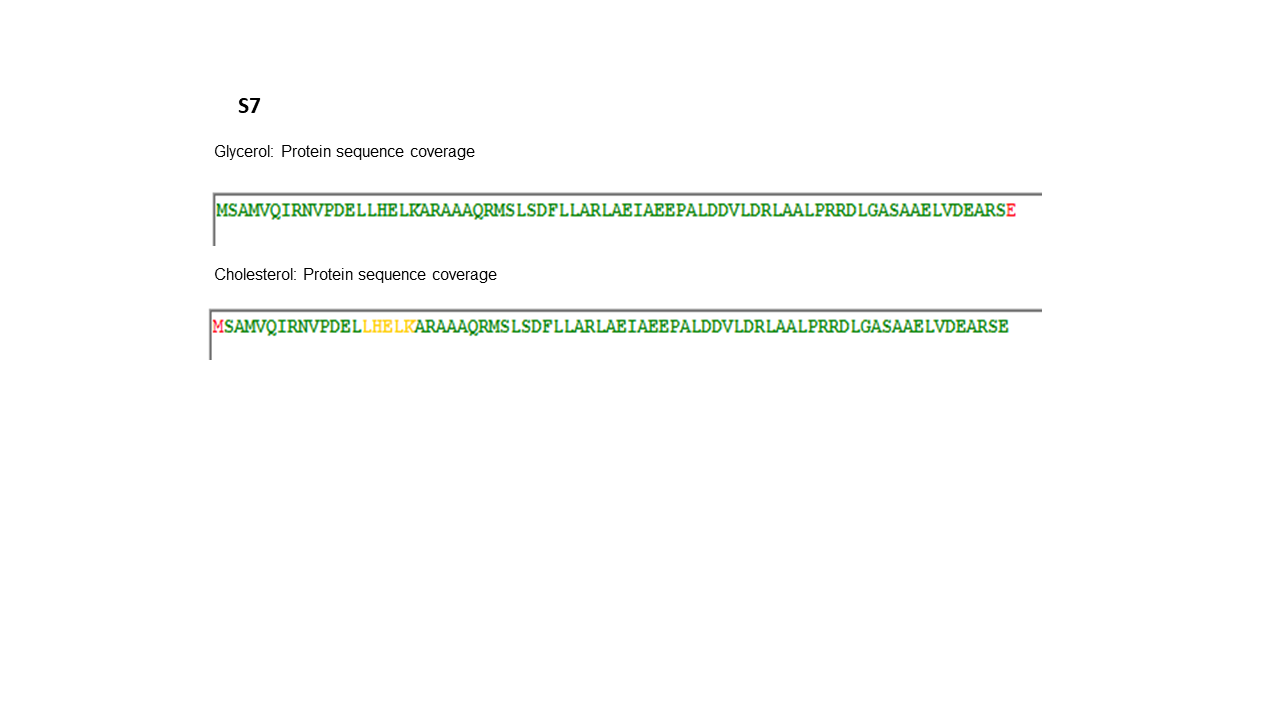

Supplement: FIG S7 [file mSystems.00855-20-sf007.tif]

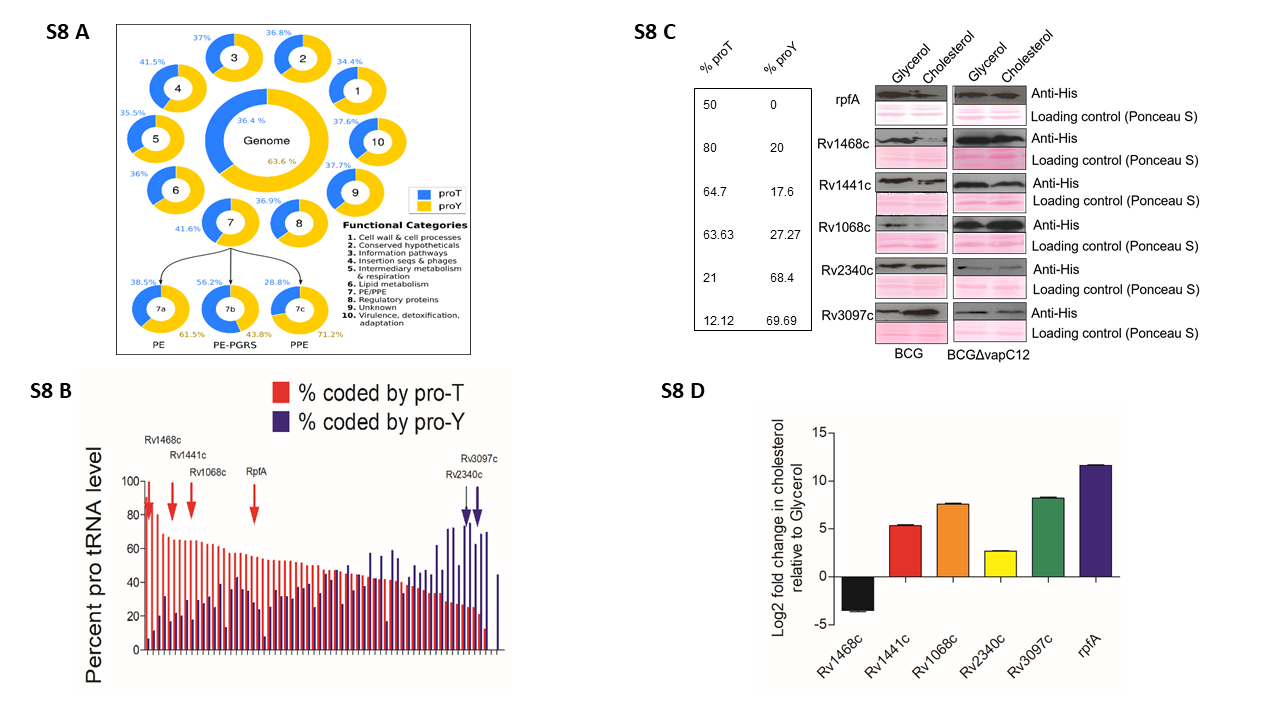

Supplement: FIG S8 [file mSystems.00855-20-sf008.tif]

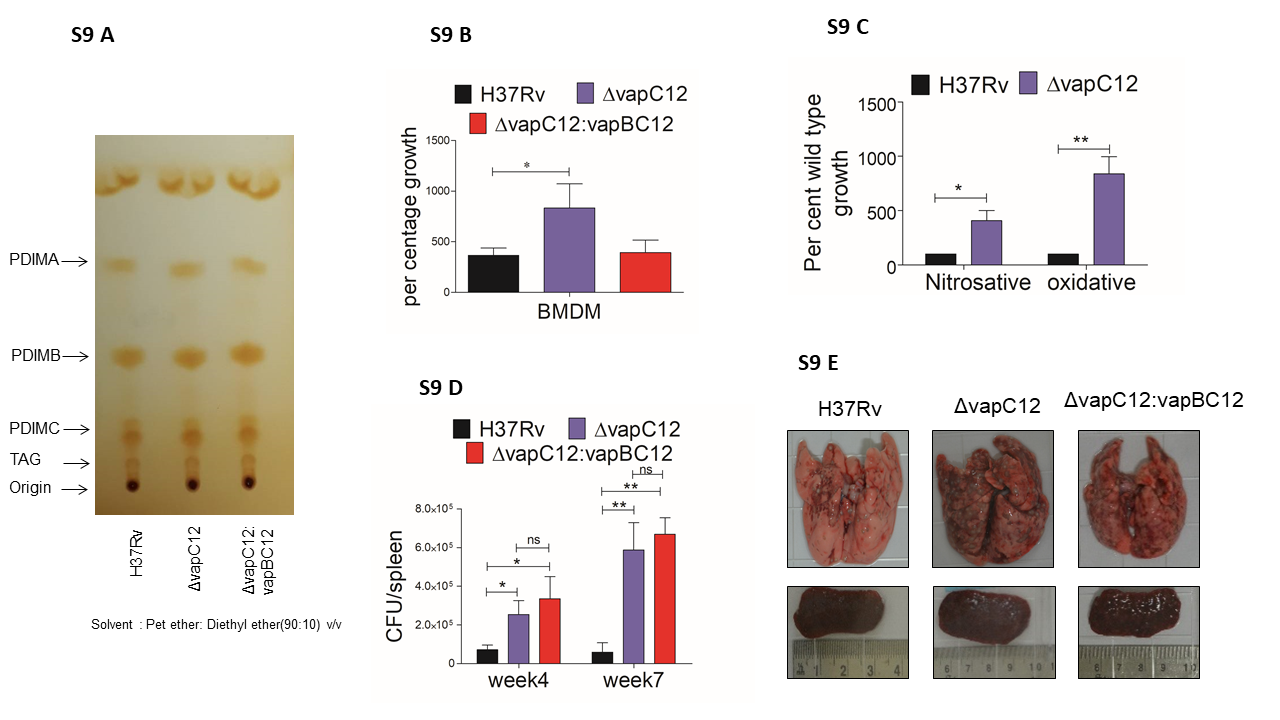

Supplement: FIG S9 [file mSystems.00855-20-sf009.tif]

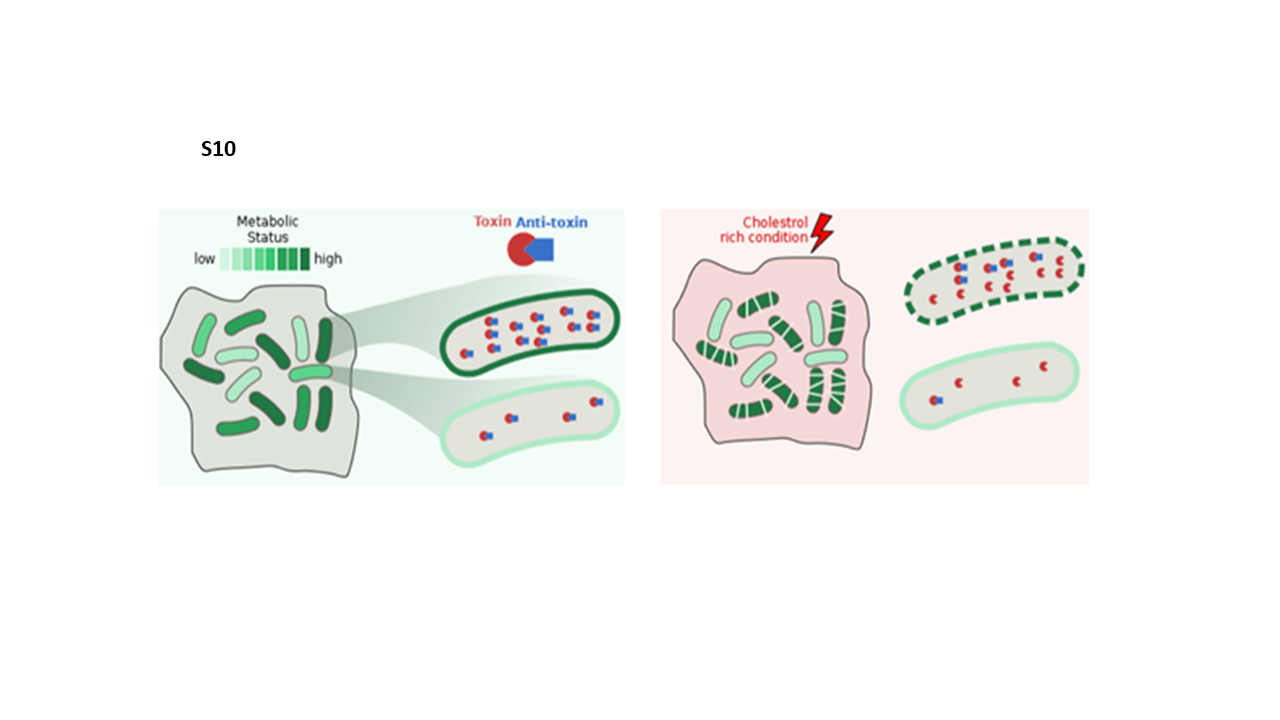

Supplement: FIG S10 [file mSystems.00855-20-sf010.tif]
